# Calibration of ionic and cellular cardiac electrophysiology models

**DOI:** 10.1002/wsbm.1482

**Published:** 2020-02-21

**Authors:** Dominic G. Whittaker, Michael Clerx, Chon Lok Lei, David J. Christini, Gary R. Mirams

**Affiliations:** ^1^ Centre for Mathematical Medicine & Biology, School of Mathematical Sciences University of Nottingham Nottingham UK; ^2^ Computational Biology & Health Informatics, Department of Computer Science University of Oxford Oxford UK; ^3^ Cardiovascular Research Institute Weill Cornell Medicine New York

**Keywords:** cardiac, electrophysiology, identification, inference, mathematical modeling, optimization, parameterization

## Abstract

Cardiac electrophysiology models are among the most mature and well‐studied mathematical models of biological systems. This maturity is bringing new challenges as models are being used increasingly to make quantitative rather than qualitative predictions. As such, calibrating the parameters within ion current and action potential (AP) models to experimental data sets is a crucial step in constructing a predictive model. This review highlights some of the fundamental concepts in cardiac model calibration and is intended to be readily understood by computational and mathematical modelers working in other fields of biology. We discuss the classic and latest approaches to calibration in the electrophysiology field, at both the ion channel and cellular AP scales. We end with a discussion of the many challenges that work to date has raised and the need for reproducible descriptions of the calibration process to enable models to be recalibrated to new data sets and built upon for new studies.

This article is categorized under:

Analytical and Computational Methods > Computational Methods
Physiology > Mammalian Physiology in Health and Disease
Models of Systems Properties and Processes > Cellular Models

## Graphical abstract

A schematic of a cardiac electrophysiology model, highlighting the components described by equations that need to be calibrated using experimental data.

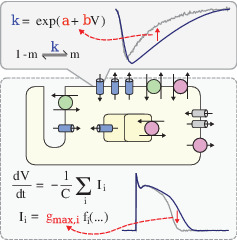

**Abbreviations**
ABC: approximate Bayesian computation
AP: action potential
APD: action potential duration
CV: conduction velocity; ERP ‐ effective refractory period
DAD: delayed after‐depolarization
EAD: early after‐depolarization
ERP: effective refractory period
hiPSC‐CM: human induced pluripotent stem cell‐derived cardiomyocyte
MCMC: Markov chain Monte Carlo
ODE: ordinary differential equation
UQ: uncertainty quantification

## 1 INTRODUCTION

Mathematical and computational modeling of cardiac electrophysiology has been one of the great success stories of systems biology, showing how ionic currents interact to produce the action potential (AP) and emergent properties in tissue, in health and disease (Winslow et al., 2011). Models are created by studying particular aspects of physiological function in great detail and then finding equations that explain (or at least reproduce) the experimental observations. This is repeated for any aspect of interest, leading to combined models that quantitatively integrate understanding gained from several experiments. The resulting models can be used to visualize processes that are not directly observable during an experiment, or to extrapolate to new situations; for example, to translate between animal models or predict the response to drugs. The usefulness of a model in these situations depends on how it was *calibrated*, which involves matching model output to data to train or learn parameter values but also comparing model outputs to validation data not used in the fitting process. How well a model can be calibrated depends both on the model itself and on the appropriateness of the experiments used to gather the data.

In this review, we discuss calibration of ionic and cellular cardiac electrophysiology models. Based on the pioneering work of Hodgkin and Huxley (1952), the first computational model of a cardiac AP was developed by Denis Noble over half a century ago (Noble, 1962). Since then, the field of mathematical and computational cardiac modeling has grown considerably, and models of cardiac electrophysiology have become increasingly detailed and complex (Noble, Garny, & Noble, 2012). The current generation of cardiac AP models integrates dynamic descriptions of intracellular ionic concentrations and secondary processes, as well as multiple, detailed ionic current formulations (see Grandi et al., 2011; Heijman, Volders, Westra, & Rudy, 2011; O'Hara, Virág, Varró, & Rudy, 2011). Such models have been applied to a variety of basic science and clinical applications, such as: investigation of fundamental mechanisms underlying initiation and sustenance of arrhythmias in the heart (Bishop & Plank, 2012; Colman et al., 2013; Qu, Weiss, & Garfinkel, 1999); assessment of genotype–phenotype relationships (Clancy & Rudy, 2001; Sadrieh et al., 2014; Whittaker, Colman, Ni, Hancox, & Zhang, 2018); preclinical drug safety testing (Davies et al., 2012; Li et al., 2017; Mirams et al., 2011); and recently to guide clinical procedures (Arevalo et al., 2016; Lee et al., 2019; Prakosa et al., 2018). Detailed reviews of the history and state‐of‐the‐art of this field are provided, for example, by Noble and Rudy (2001), Rudy and Silva (2006), Fink, Niederer, et al. (2011), Winslow et al. (2011), Noble et al. (2012), and Heijman, Erfanian Abdoust, Voigt, Nattel, and Dobrev (2016). Although we focus specifically on electrophysiology, similar principles apply in modeling cardiac mechanics—see Niederer, Campbell, and Campbell (2019) for a recent review of such models.

We start by providing a definition of calibration that we will use throughout this review.

### 1.1 What is model calibration?

Model calibration is the process of adjusting parameters of a model to maximize the agreement between observed data and simulations. This is an indispensable component of model development. Examples of when calibration is necessary are (a) creating a new model, (b) adapting an existing model to new contexts (including personalization/tailoring to subgroups of the population), and (c) studying the effects of an experimental intervention, a mutation, or a drug, which may involve reparameterization and/or the addition of extra equations. In this article, we focus primarily on estimating parameters for existing mathematical models, rather than deriving the underlying equations, which we will discuss later in the Challenges section.

### 1.2 What needs to be calibrated?

**Figure 1:**
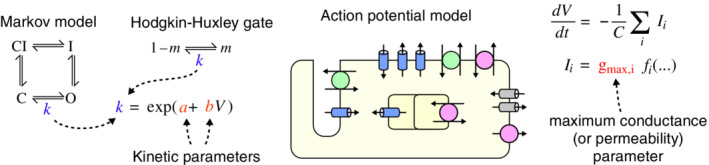
Cardiac electrophysiology models and parameters to calibrate. Left: currents through ion channels or active pumps are modeled using Markov or Hodgkin–Huxley models which contain several reaction rate coefficients. These rates determine the current *kinetics*, and finding the parameters of these rates is the subject of Section 3. Right: an action potential model contains (among other things) submodels for its ion channels, pumps, and transporters, and the magnitude of each current type depends on a parameter for its maximum conductance or permeability. Section 4 deals with the problem of setting these parameters

Some of the parameters within cardiac electrophysiology models which need to be calibrated are illustrated in Figure 1. First, in Section 3, we discuss calibrating models of the currents through ion channels and pumps/transporters. In particular, we focus on ion‐channel models represented either as a set of Markov transitions between states (similar to a kinetic scheme in physics or chemistry) or as a Hodgkin–Huxley model (a series of independent two‐state reactions called “*gates*”). Both model types contain several voltage‐dependent reaction rates, and it is the parameters of these rate equations (the “*kinetic parameters*”) that need to be estimated.

In Section 4, we discuss combining multiple ion current models into a model of the cardiac AP. The interaction of the currents passing through cellular membranes determines the shape of the AP, the calcium transient and subsequent cell contraction. These processes are controlled by a maximal current density parameter (a “maximal conductance” or “maximal permeability”) for each current in the model. The values of these parameters vary between cell‐types (and within cells of a given type) and are related to membrane expression levels of the corresponding channel/pump/transporter protein(s). Many models also contain subcellular Ca^2+^ components with parameters which require calibration, related to microanatomical structure within cells (the sarcoplasmic reticulum, *t*‐tubules, and microscopic spatial domains between and within these structures).

In this review, we will highlight classic and recent publications on model calibration and experimental design in cardiac cellular electrophysiology. In the final sections, we give tips for avoiding common pitfalls and discuss open challenges in the field. Before discussing examples of model calibration and related advances, we first introduce a number of concepts that are important to consider.

## 2 CONCEPTS

### 2.1 Context of use

The *context of use* of a model is the conditions under which the model will be used. This context of use should be considered at the outset, as it has significant consequences for the approach to model calibration.

It may be that the primary purpose of a modeling study is to identify a plausible biophysical mechanism. In this case, the context of use would be limited to qualitative fits, and it may be perfectly adequate to find “one plausible parameter set that can fit the data.” In other studies, we want to make quantitatively accurate predictions which are sensitive to particular parameters and use a model in a safety‐critical setting, to guide treatment for instance. In this case, the context of use dictates that we should take into account our uncertainty in the parameters, assigning probabilities or a range of different model predictions as a result, and test the model in new situations that might occur in new patients. Context of use is explored further in Pathmanathan, Gray, Romero, and Morrison (2017), who suggest a careful audit of how well a model's testing regime matches the context of use.

Given that there are typically many models which represent the same biological system, the context of use will also inform the model choice. For instance, simple models may not be capable of reproducing delayed after‐depolarizations (DADs, Fink, Noble, & Noble, 2011), and thus by definition are unsuitable for investigating mechanisms of DAD initiation at the cellular level. In contrast, complex models may offer no advantage for the simulation of spiral waves in tissue, and so simpler, more easily parameterized models might be used instead (Bueno‐Orovio, Cherry, & Fenton, 2008; Fenton & Karma, 1998). Using models in inappropriate or unanticipated contexts can highlight significant model inadequacies (Noble et al., 2012), and thus the issue requires careful consideration to avoid drawing erroneous, model‐dependent conclusions.

### 2.2 Interpolation, extrapolation, and overfitting

Scientific insights in cardiac electrophysiology are usually extrapolation almost by definition—for example, an experiment is performed in an animal with the aim of gaining insights relevant to humans. Even when using human cells, the best‐case scenario would be that findings are extrapolated from one cell to another, or from one patient's heart to another's. In the scenario where the context of use is very similar to that in which the model was trained we might be interpolating behavior between areas where it is well fitted. In this case, a statistical approach to model building (regression or curve fitting) may suffice to make good predictions.

**Figure 2:**
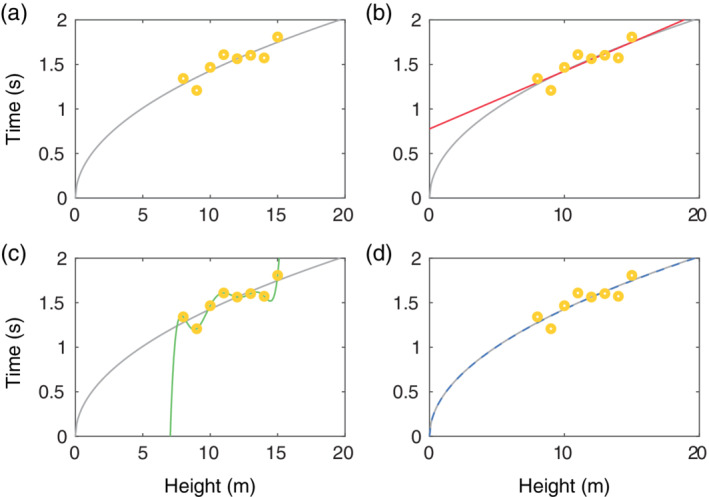
A simple example illustrating interpolation, extrapolation, and overfitting. (a) The gray line in all plots is $t=\sqrt{2y/g}$, the time it would take a mass to fall a distance *y* from rest under gravitational acceleration *g* = 9.81 m/s^2^ (with no air resistance). The yellow circles denote observations we might make when dropping a mass from different heights up a tower if there were normally distributed error in timing the process with a stopwatch (*σ* = 0.1 s in this case). (b) If we were taking a purely statistical approach we might fit the red straight line through these data. This line would be reliable for interpolation between the measured yellow data points, but bad for extrapolation outside them (the red line predicts it would take 0.78 s for the mass to fall no distance at all at height = 0). (c) We could fit a polynomial spline to the data, shown in green. This curve goes through all eight data points exactly (in this case the polynomial is of degree 7, with eight parameters), but by doing so we are fitting the noise in the data and this line is less reliable for interpolation, and very bad for extrapolation, even compared to the straight line in (b). (d) The blue dashed line is what we get when fitting *g* in the (correct) mechanistic model $t=\sqrt{2y/g}$, in this case a best fit (least squares) returns *g* = 9.84 m/s^2^ rather than 9.81 m/s^2^ due to the noise on the data, but this would still provide reliable interpolation and extrapolation for many contexts of use

In Figure 2, the results of a simple thought experiment are shown. Figure 2 illustrates why biophysical models can be preferable over a statistical or machine learning approach when the context of use involves extrapolation. In the absence of having data available for every scenario of interest, the physical laws and mechanisms within the biophysical model allow for reliable extrapolation (Panel d), provided they are represented accurately enough. In the example given in Figure 2 the spline model (Panel c), which has the best fit to the data, is the worst in terms of predicting the “true” process shown by the gray line across the whole axis. The dangers of underfitting (Panel b) and overfitting (Panel c), are commonly acknowledged as an issue for statistical regression and machine learning models—picking the appropriate complexity is subject to what is known as the “bias‐variance tradeoff” (Hastie, Tibshirani, & Friedman, 2003).

Overfitting may seem less of an obvious issue for mechanistic models. However, adding extra gates or states to an ion‐channel model or extra currents/subspaces to an AP model in an attempt to better fit the data risks overfitting the complexity of a mechanistic model, with the same overall effect as seen in Figure 2c. For this reason, a reasonable fit with a simple model may be a better representation of the underlying biology (and more reliable for extrapolation and prediction) than a more complex model that fits the data better.

Assessing the reliability of a model's predictions, and guarding against overfitting, is commonly done by separating the calibration process into training and validation.

### 2.3 Training and validation

Calibration is typically defined as the process of adjusting parameters of a model to maximize the agreement between observed data and model simulations (Gábor & Banga, 2015; Read, Alden, Timmis, & Andrews, 2018; Stadtländer, 2018)—although “simulations” is often used interchangeably with “calculations,” “outputs,” or “predictions.” In our introduction, we avoided the use of the word “prediction” in our definition, in order to draw a careful distinction between what is truly a prediction (emergent behavior due to the model representing the system well) and what agrees *by design* in the calibration process. For instance, in the example shown in Figure 2 our training regime consisted of data points in the range 8–15 m. As such, we would not call model simulations within this range “predictions,” as they are based on interpolation of model outputs that match training data well by design. Above or below this range we are making extrapolations and predictions for which we have not yet seen any data. Measuring the behavior at an extrapolated height of 0–5 m in this case would highlight whether the straight line, polynomial spline, or mechanistic models is most suitable, and would be a good test for the validity of the models (*validation data*).

A conventional statistical model building exercise would suggest that the training and validation regimes should be very similar to both each other and the context of use. When building biophysical models we may want to “challenge” our mechanistic assumptions by performing validation in a very different regime to the training data, to test whether our biophysical assumptions hold and assess whether we have described the mechanisms well. In either case, when returning to using models for predictive purposes, it makes sense for a validation regime to be similar to the context of use to give the best evaluation of predictive power in that setting.

In Figure 2, it was easy to see that we were interpolating between 8 and 15 m and extrapolating outside. But in general, for validation predictions to gain confidence in the mechanistic aspects of the model, we are interested in changing the scenario in more complex ways. It is not always easy to establish whether we are in an “interpolation” or an “extrapolation” setting. For instance, a model might have been trained to replicate 1 Hz AP traces. It would seem reasonable to say that simulations at 2 Hz would be extrapolation and therefore good validation—simulations matching 2 Hz data provides some evidence that the underlying biophysics have been captured well. But is that true? Would simulations at 1.5 Hz be predictions? 1.25 Hz? 1.001 Hz? The boundary between interpolation/training and extrapolation/validation is not easy to define in most situations.

Another important consideration comes when making any further decisions based on a validation exercise. For instance, one might run a number of possible models and choose the one that worked best in validation, or pick a simulation output threshold to make a decision based on optimal validation performance. These are very sensible things to do, but beware: doing this turns the model “validation data” into “training data” for model selection or the decision threshold! Subsequent conclusions are vulnerable to the same weakness as only evaluating performance on training data; namely that we select the thing that happened to work well for this dataset but which does not generalize to new data. In the machine learning community this is addressed by keeping a third hidden *test* set (in addition to training and validation) which one can then use to evaluate the performance of the chosen model after training and validation. See Hastie et al. (2003, Chapter 7) for an accessible introduction to this important idea.

### 2.4 Statistical noise models

Fitting parameters by running an optimization is preferable to manually “tweaking” parameters in terms of reliability and reproducibility. Parameter estimation can be considered in the context of minimizing an objective function that describes an error, that is, between experimental and simulated data; but can also be seen as maximizing a likelihood, that is, finding the model parameters that are most likely to give rise to the observations.

When the statistics literature refers to a “model” it typically relates to parameters of a distribution or regression curve. In mechanistic modeling settings it is helpful to think of a “data‐generating process” which is composed of both a mechanistic model and a noise model, for example,

(1) Experimental observation=Mechanistic model output+Noise model.

The noise model is usually stochastic. A common choice is independent and identically distributed (i.i.d.) Gaussian noise, often with mean zero (i.e., independent samples $\sim N\left(0,\sigma^{2}\right)$ with the same *σ* on each time point in a time series). The mechanistic model is often deterministic, in the cardiac setting usually the solution of a system of ODEs; although it could also be stochastic, for example, if stochastic differential equations were to be used to represent the opening and closing of individual ion channels. The additive noise model shown in Equation (1) is common, but more exotic alternatives and combinations of the mechanistic and noise terms are worth investigating in cardiac applications.

One consequence of writing a data‐generating model like this is that one can infer parameters for both the mechanistic model and the noise model at the same time, commonly by writing a *likelihood function* for the experimental observations given the parameters within both the mechanistic and statistical models, and then maximizing this likelihood (using numerical optimization) to find all of the parameters at once.

Another advantage of the statistical approach is that it offers a natural way to incorporate prior knowledge about the parameter values into the final estimate: If a *prior distribution* on the parameters can be defined, it can be combined with the likelihood function through *Bayes' rule*, to obtain a function for the *posterior distribution* (which incorporates both prior knowledge and what was learned from this experiment). We can then use optimization to find the parameter set at the maximum of the posterior distribution, but it is also possible use sampling methods such as Markov chain Monte Carlo (MCMC) to approximate the shape of the whole posterior distribution. For an introduction to Bayesian methods, see, for example, Gelman et al. (2013) and Lambert (2018).

### 2.5 Uncertainty and variability

It is important to distinguish between parameters in biological models varying, being uncertain, or both. Biological variability is ubiquitous as systems respond to the different environments in which they operate and the particular genetic make‐up of individuals. Parameters taking distinct values in different individuals (people or cells) within a population is termed *extrinsic* variability. Various statistical approaches exist to deal with extrinsic variability in parameters and describing how they are distributed across a population—the primary approaches are called “mixed effects models” in a frequentist framework (widely used in studies of drug absorption and metabolism in patient populations, see Owen and Fiedler‐Kelly (2014) for a textbook introduction) or “hierarchical models” in a Bayesian framework (see Gelman et al., 2013).

It is important to distinguish such variation from parameters simply being uncertain due to lack of information about them in measurements (Mirams, Pathmanathan, Gray, Challenor, & Clayton, 2016)—that is, we do not know what particular values a parameter takes because there are a wide range of possibilities consistent with the data. In essence, there is a difference between whether or not something can be measured and whether it varies in reality. The degree to which we find ourselves in the “lack of information” situation can be examined using the concept of identifiability.

### 2.6 Identifiability

A working definition of identifiability is that “only one small region of parameter space gives the optimal fit to the data” as opposed to “parameter values can take a wide range of values and be equally consistent with the data.” Or in other words, there is a small global minimum in parameter space for the function we optimize when calibrating parameters. Note that identifiability is a statement about both a particular model's parameters *and* a particular experimental observation used to calibrate them. If the outcome of a calibration exercise results in unidentifiable parameters, or combinations of parameters, this is not necessarily a problem. A given prediction in a particular context of use may not be sensitive to the precise values of the poorly constrained parameters and wide ranges may still result in well‐constrained predictions. Such behavior has been termed *sloppiness* and it has been suggested that it is a “universal” property of systems biology models (Brown & Sethna, 2003; Gutenkunst et al., 2007; Waterfall et al., 2006). A counterpoint was raised by Apgar, Witmer, White, and Tidor (2010) who pointed out that this sloppiness is unidentifiability which can be remedied by performing different experiments. In fact, many classical optimal experimental design criteria are based on reducing the same quantities used to define sloppiness; we will discuss experimental design in Section 2.6.3.

#### 2.6.1 When is unidentifiability a problem?

Unidentifiability becomes a problem when new predictions from a model are strongly dependent on the particular values of parameters that were unidentifiable when the model was fitted.

**Figure 3:**
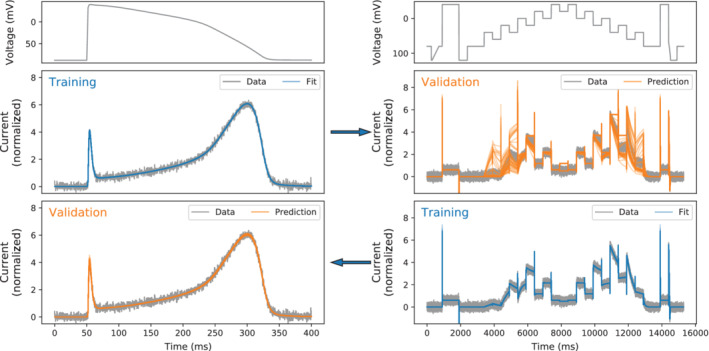
Consequences of unidentifiable parameters. Top: two voltage‐clamp protocols—(Left) an action potential clamp and (Right) a “staircase” clamp from Lei, Clerx, Beattie, et al. (2019). In this example we generate synthetic data under these protocols (shown with a gray jagged line) using the ten Tusscher, Noble, Noble, and Panfilov (2004) *I*_Kr_ model, saying for this example that this model with its published parameters represents the “ground truth.” Twelve kinetic parameters were set as per the original paper values (and conductance was fixed to one for simplicity), then a realistic amount of Gaussian noise ($N\left(0,0.15\right)$) was added to represent a whole‐cell patch‐clamp expression system experiment. Middle: the currents that result from the voltage‐clamp protocols above: (left) in blue we show 45 of the best fits to the action potential clamp that we achieved from different initial guesses, all of these fits are closely overlaid and appear to be excellent. (Right) we use these parameter sets to make predictions of staircase protocol currents, shown in orange. The simulations diverge wildly from the data and often make very bad predictions in this new setting. Bottom: (right) we perform the opposite procedure and fit the 12 kinetic parameters to the staircase protocol current, in this case all 45 fits (blue) are tightly constrained and indeed return almost the same parameter set (close to the one that generated the synthetic data). (Left) predictions from these fits (orange) are all excellent for the action potential clamp. This example is based on one from Fink, Niederer, et al. (2011)

As an example, in Figure 3 we show the result of fitting 12 *I*_Kr_ model kinetic parameters from ten Tusscher et al. (2004) to the *I*_Kr_ provoked by clamping to an AP waveform, which in this case is “*synthetic data*” generated using the parameter values in the original paper. We repeat the fitting multiple times from different initial parameter guesses (uniformly sampled from a modest 0.66 to 1.5× the literature values), and plot the 45 best fits in Figure 3. All of these 45 parameter fits are excellent (what would appear to be a good minimum has been found in all cases, the fits to the data look perfect), but there is no global minimum as the parameter sets we obtain differ substantially. For this example we used a global optimization algorithm (CMA‐ES; Hansen, 2006), but similar results are seen with simpler ones such as a simplex algorithm (Nelder & Mead, 1965). For recent comparisons of global optimization methods in systems biology, see Banga (2008), Raue et al. (2013), Degasperi, Fey, and Kholodenko (2017), and Schälte, Stapor, and Hasenauer (2018). As the fits could not be any better in this case, the optimizer choice does not influence the behavior we observe. If we then predict the *I*_Kr_ provoked under a different protocol, we get substantial divergence from the synthetic data and make predictions with very large errors (often larger errors than the current itself). However, if we perform the procedure by fitting to the more complicated protocol then all 45 fits obtain parameter sets close to the original ones (find the global minimum), which then predict behavior under the AP clamp excellently. So here the AP clamp results in unidentifiable parameters, and the staircase clamp results in identifiable parameters (for this model's kinetic parameters). Assessing whether we have experimental data which allows us to identify all parameters is therefore a task of primary importance.

#### 2.6.2 Methods to assess identifiability

There are various methods to assess identifiability. *Local identifiability* methods typically calculate a sensitivity matrix (partial derivatives of observations with respect to parameters) or the related Fisher information matrix (Cobelli & Distefano, 1980). Conclusions are drawn about whether all parameters are identifiable by looking at the rank of the matrix (applied in a cardiac setting by Fink and Noble, 2009) or other properties indicating the linear independence of the parameters' influence on the outputs (Sher et al., 2013). The drawback of local methods is that the parameters of interest need to be known to estimate whether they are identifiable, clearly not the case if we need to calibrate them(!), and there are various algorithm tolerances to choose in terms of “how independent” parameters need to be. However, useful information can still be gained by examining local identifiability at an initial guess and the final calibrated sets of parameters.

*Structural identifiability* methods can assess identifiability globally (for any parameter set) using algebraic techniques to examine the analytic sensitivity of outputs to various parameters/inputs, see for instance Villaverde, Barreiro, and Papachristodoulou (2016). Such methods can become difficult to apply to differential equation models where outputs are typically integrals over time, but Villaverde, Evans, Chappell, and Banga (2019) apply them to a simple ion‐channel example.

*Practical identifiability* is a pragmatic approach where synthetic observable data is generated with a range of possible model parameter sets, and one then evaluates whether the parameter sets can be recovered using the proposed calibration scheme. This can be repeated optimization from different initial guesses, a brute‐force “grid search” of the objective in low enough dimensions, or MCMC based explorations of an objective function (see Hines, Middendorf, & Aldrich, 2014; Siekmann, Sneyd, & Crampin, 2012). The example we showed in Figure 3 was actually a practical identifiability assessment for one parameter set: fitting to current from an AP clamp is unidentifiable, but fitting to the staircase protocol is identifiable for the given *I*_Kr_ model. Finding practical identifiability guarantees there is structural and local identifiability in the parameter regime of interest. So while practical identifiability is something of a brute‐force approach, we highly recommend the procedure as cardiac simulations are typically computationally lightweight enough to permit it.

The concept of identifiability being equal to a narrow region of plausible parameter space can also be quantified using the notion of information content.

#### 2.6.3 Protocol information content and protocol design

Experimental protocol design can be performed algorithmically using “Optimal Experimental Design” (Chaloner & Verdinelli, 1995; Ryan, Drovandi, McGree, & Pettitt, 2016). Experiments or protocols are optimized in respect to some criterion, such as a protocol designed to maximize sensitivities of model outputs to the parameters in some sense. Such protocols may offer advantages over conventional protocols used in physiology experiments, in that they can be designed with the specific aim of parameter estimation, or model calibration.

Loosely speaking, a protocol's information content can be quantified in terms of how much it reduces our uncertainty in parameter values. This reduction of uncertainty is a clear target for experimental design—a protocol giving identifiable parameters will be selected over one that does not. The now traditional optimal experimental design criteria based on properties of a sensitivity or information matrix do precisely this (see Smucker, Krzywinski, & Altman, 2018, for an accessible introduction), but are based on local sensitivity for a best set of parameters. In a Bayesian framework, reducing uncertainty becomes narrowing the posterior distribution for parameters, as discussed in a systems biology context by Liepe, Filippi, Komorowski, and Stumpf (2013). A global algorithm such as this potentially offers benefits over the classical local methods, by exploring all the relevant parameter space, particularly for nonlinear models.

All of these methods require some assumptions about parameter ranges and distributions in order to design the best experiments to measure them. This inevitably leads to iterations of experimental design and experiments to optimize the design to measure the parameters of the particular system. Performing these iterations quickly is a challenging but attractive goal. It may even be possible to perform the experimental design online in real time during one experimental run for things such as measuring drug action on a well‐characterized ion current, or measuring maximal current densities in a single cell.

### 2.7 Verification, validation, and uncertainty quantification

Before using models to make safety‐critical quantitative predictions, the engineering community commonly applies the verification, validation, and uncertainty quantification principles (US National Research Council, 2012). Verification relates to verifying that the simulation is performing correctly: that there are no bugs in implementation, and equations are being solved accurately. This verification step was discussed in a cardiac simulation context by Pathmanathan and Gray (2013). Validation refers to the complex task of assessing the validity of a model's predictions for a given context of use, which is discussed in Pathmanathan and Gray (2018). Uncertainty quantification (UQ) refers to the process of characterizing any uncertainty in prediction‐specific inputs (or parameters) and propagating this uncertainty through a simulation to build a probability distribution of possible outputs. Some of the first UQ work performed in a cardiac setting was by Elkins et al. (2013) for a distribution of drug block parameters and Pathmanathan, Shotwell, Gavaghan, Cordeiro, and Gray (2015) for sodium current inactivation parameters.

## 3 ION CURRENT MODELS

Models of the cardiac AP are composed of several submodels, describing particular ionic currents through channels and transporters, diffusive fluxes between intracellular compartments, and calcium buffering and release in the sarcoplasmic reticulum. Of these components, the voltage‐sensitive passive ion‐channel currents were the first to be modeled, and the problem of fitting mathematical models to electrophysiology data from “whole‐cell voltage‐clamp” experiments has been particularly well studied in cardiac, neuronal, and general excitable‐cell physiology.

### 3.1 Voltage‐sensitive ion channels

**Figure 4:**
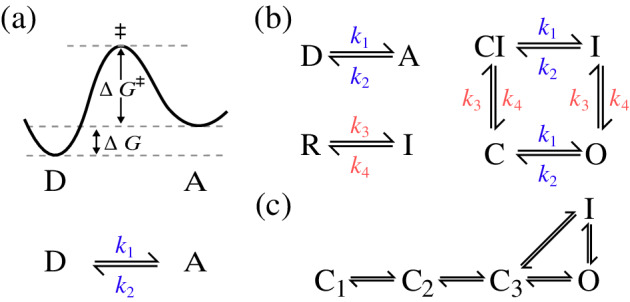
(a) An energy barrier model of a channel with two stable states: activated (A) and deactivated (D). This leads naturally to a reaction scheme as shown below, with reaction rates *k*_1_ and *k*_2_. (b) Adding a second, independent, transition leads to a two “gate” Hodgkin–Huxley model including an inactivated (*I*) and recovered (*R*) state. The equivalent Markov model representation is shown to its right. (c) Markov models are a more general class of models which would allow transitions between any different states

While ion channels are massive macromolecular complexes (Abriel, Rougier, & Jalife, 2015), their voltage‐sensitive opening and closing can be reasonably well described as a series of transitions between a small number of discrete states (Sigg & Bezanilla, 2003; Silva et al., 2009; Sterratt, Graham, Gillies, & Willshaw, 2011; Tsien & Noble, 1969). Models of ionic currents based on this idea are known as “Markov models” in electrophysiology, and more generally as “kinetic schemes.” Hodgkin–Huxley (HH) type current models can be viewed as a subclass of Markov models, and consist of independent pairs of states (known as “gates,” see Figure 4), all of which need to be open for current to flow. In both cases, the transition rates can be modeled using transition‐state theory (Sigg & Bezanilla, 2003; Tsien & Noble, 1969; i.e., with the Eyring equation), although equations without a physical basis have also frequently been used.

A simple model of an ionic current assumes that only a single species of ion passes through the channels, and that the driving force for the ion transfer is the difference between the membrane potential (*V*) and the reversal potential (Nernst potential) for the ion (*E*). Application of Ohm's law then leads to the following equation for current:

(2) $$I=g_{\max}\cdot g\left(V,p\right)\cdot \left(V-E\right),$$

where the maximal conductance or current density *g*_max_ is determined by the number of channels and the conductance of each individual channel, while the fraction of conducting channels is given by *g*(*V*, *p*). The parameters *p* in *g*(*V*, *p*) (the “kinetic parameters”) will be the main focus of this section, but inferring them typically requires simultaneous inference of *g*_max_ and *E*, although the latter can also be calculated from the ionic concentrations on either side of the membrane for most currents. Other formulations for the ionic current which are commonly used in cardiac modeling include the Goldman–Hodgkin–Katz formulation (see Keener & Sneyd, 2009, Chapters 2 and 3 for more details). In this case, there is still the concept of a maximal current density and gating kinetics but a different expression for the “driving force.”

#### 3.1.1 Steady states and time constants

Several studies have focused on the problem of estimating steady‐state currents and time constants of current decay/activation from whole‐cell current recordings at particular voltages. While these analyses are often performed on synthetic data and can be highly mathematical, their results are relevant to experimenters as well as modelers, as time constants and steady states are still commonly used to report experimental results (although, for reasons that will become clear, we implore any experimenters who are reading to please *also* publish the current time courses in a digital format).

**Figure 5:**
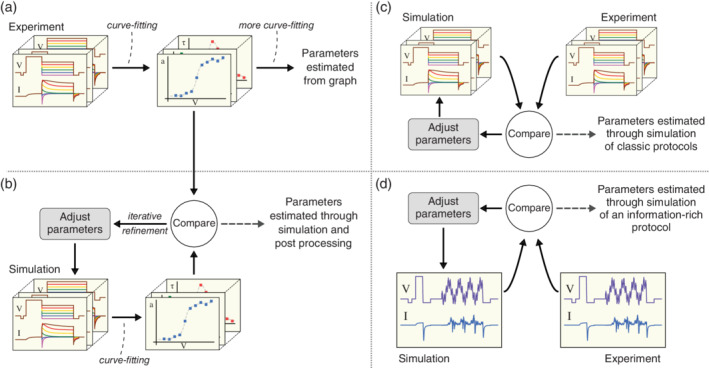
Four ways of fitting a voltage‐dependent ion‐channel model to whole‐cell current experiments, as described in Clerx, Beattie, et al. (2019). (a) Method 1. Currents are measured using classical voltage‐clamp protocols and analyzed to obtain time constants and steady‐state (in)activation levels for several voltages. Next, curves are fit to the obtained points and inserted directly into the model. (b) Method 2. Applying the same voltage‐clamp protocols in a simulation leads to a simulated set of currents, that can be similarly processed to obtain simulated (in)activation steady states and time constants. Next, an error measure is defined between the simulated and experimental summary data points, and minimized using numerical optimization. (c) Method 3. Like Method 2, but now the error measure is defined directly on the current traces, eliminating the need to perform processing to generate summary curves. (d) Method 4. Like Method 3, but using a single condensed voltage‐clamp protocol instead of a series of classical protocols

Many published studies consider the specific problem of fitting a two‐gate Hodgkin–Huxley model (i.e., with an activation and an inactivation gate). Such models can be written in a form where each gate's kinetics are described by a voltage‐dependent steady‐state and time constant, and the fitting procedure involves extracting these quantities from current measurements *independently for several voltages*. Detailed examples of these analysis methods are given in the supplementary materials to Clerx, Beattie, et al. (2019). A model “fit” is then made by fitting (often somewhat arbitrary) curves through the resulting points, and inserting the resulting equation directly into a model. A schematic overview of this procedure is shown in Figure 5a.

A crucial point regarding this method was made by Beaumont, Roberge, and Leon (1993), who showed that the steady states can only be measured correctly if the time constants of the different gates (e.g., activation and inactivation) are “well‐separated.” In other words, our ability to measure steady states of activation and inactivation at any voltage *V*, depends on there being a large difference in the time constants of activation and inactivation at that voltage. The same conclusion was reinforced by Willms, Baro, Harris‐Warrick, and Guckenheimer (1999) and Lee, Smaill, and Smith (2006). Having well‐separated time constants often means that one of the processes is either very fast (e.g., *I*_Na_ activation) or very slow (e.g., *I*_Kr_ activation). There is an interesting experimental consideration here, in that very fast processes are difficult to measure and may require specialized equipment (Sherman, Shrier, & Cooper, 1999), while for slow processes great care must be taken that the voltage steps are long enough (Clerx, Beattie, et al., 2019; Vandenberg et al., 2012), which poses difficulties if cells becomes unstable during measurement. It is preferable not to have to wait for steady states to arise to fit a model's parameters.

#### 3.1.2 Hodgkin–Huxley model parameters

A second paper by Beaumont, Roberge, and Lemieux (1993) investigated the problem of finding the steady states of a HH model with nonseparable time constants. As part of their solution, which they termed an *inversion procedure*, they used a Boltzmann‐curve to connect the steady states at all measured voltages (which provides a stronger constraint on the data than fitting each voltage independently) and then fitted it using the peak current and the time‐to‐peak, reasoning that these points provide the highest signal‐to‐noise ratio. The final parameters are then obtained using numerical optimization. A further extension of this method was presented in Wang and Beaumont (2004), which avoids numerical optimization, but requires an updated voltage‐step protocol that is not well tolerated by all cell‐types. The fourth paper in the series (Raba, Cordeiro, Antzelevitch, & Beaumont, 2013) provided an experimental confirmation of the need for an updated experimental protocol, and contained experiments on synthetic data showing an improved version of the Wang and Beaumont (2004) method.

Willms et al. (1999) and Lee et al. (2006) performed similar analyses of two‐gate HH models, and both showed that estimating steady states and time constants independently (or “disjointly”) leads to an error in the estimate of steady‐state activation that scales with the ratio of the time constants of activation and inactivation. The work by Lee et al. (2006) went on to recommend an approach that minimizes an error in peak currents, time‐to‐peak, and steady‐state currents, while Willms et al. (1999) recommended fitting current traces directly (see Figure 5c). Both studies used simulations to show the superiority of their methods over a conventional approach.

A different approach was taken by Csercsik et al. (2010) and Csercsik, Hangos, and Szederkenyi (2012), who investigated the theoretical identifiability of steady states and time constants from a single voltage‐step. For HH models with two gates, each with Exponent 1, they were able to show that time constants are identifiable, while the maximal conductance and steady states form an unidentifiable pair. This analysis was extended to general HH models (any number of gates with any exponents) by Walch and Eisenberg (2016), with the additional result that the exponents themselves are identifiable. This is valuable work for the analysis of single voltage steps, but the analysis has yet to be extended to multi‐voltage‐step protocols, where we know from practical identifiability experiments that knowledge of the relationship between parameters at different voltages can provide full identifiability of parameters (e.g., by assuming voltage‐dependent rates of the form shown in Figure 1).

#### 3.1.3 Markov models and optimization approaches

Many models postulate a more complicated kinetic scheme than independent HH gates, so that the system can no longer be described by a set of independent time constants and steady states. Instead, these models are fit by defining an error measure between experimental data and simulations using some initial guess for the parameters, and then iteratively adjusting the parameters until the error is minimized. Unlike the methods discussed in the previous sections, which are restricted to HH models, optimization‐based methods can be used for any model of an ionic current.

A common method to use optimization for model calibration, is to simulate conventional voltage‐clamp protocols, calculate steady states and time constants (temporarily assuming a HH formalism), and then adjust parameters until the difference between simulated and experimental values is minimized (see Figure 5b). This method has the advantage that it requires only steady states and time constants as input, which are easy to find in the experimental literature, but it can lead to large errors and an unnecessarily complex fitting procedure (Clerx, Beattie, et al., 2019).

An alternative method, shown in Figure 5c, is to compare the recorded and simulated currents directly. An early example of this method was shown in Balser, Roden, and Bennett (1990), who simulated channel open probability in a three‐state Markov model (with Eyring rates) and fitted this to the estimated open probability from macroscopic whole‐cell recordings. They showed the applicability of their method to measurements in Guinea pig ventricular myocytes, but also verified its performance on a simulated set of 90 virtual “cells.”

Identifiability of Markov model parameters has focused on *local* analysis; which starts from a known solution in parameter space. If the model is found to be unidentifiable at this point this proves a lack of global identifiability, but no guarantees can be given for the global identifiability of locally identifiable results. Several models and voltage protocols were tested for local identifiability by Fink and Noble (2009), who used it to highlight several unidentifiable parameters in published Markov models. An extension of this method based on singular value decomposition was later presented in Sher et al. (2013) and used to create reduced models by eliminating redundant parameters. Note that even a practical identifiability analysis based on global optimization is still local with respect to the single set of parameters that generated the synthetic data, and so repeating these exercises with multiple possible parameter sets is also recommended.

#### 3.1.4 Protocol design

Practical identifiability analysis has been used as a tool to design voltage‐clamp protocols. For example, Fink and Noble (2009) analyzed and shortened popular voltage‐clamp protocols by removing steps that did not affect parameter identifiability. Zhou et al. (2009) also tested various protocols for identifiability before performing experiments, and the work on HH model identifiability by Csercsik et al. (2012) ends with several recommendations for protocol design.

A more radical approach to protocol design had previously been taken by Millonas and Hanck (1998b), who measured *I*_Na_ currents resulting from protocols that fluctuated rapidly between a high and a low voltage and used it for model calibration. They then fit a model to currents from conventional voltage‐clamp protocols, and showed that it could not predict the response to the novel protocol, showing that the rapidly fluctuating protocol had the potential to uncover new information (Millonas & Hanck, 1998b, 1998a). A follow‐up study (Kargol, Smith, & Millonas, 2002) investigated systematic protocol design, and showed an example where a protocol was designed to maximize the predictions from two competing models that showed a similar response to conventional protocols. One method to systematically generate (and compare) protocols, is to create them by superimposing similar waveforms, for example, sine waves of different frequencies and amplitudes, and the authors briefly discuss this before suggesting *wavelets* as a more suitable basis (which they further explored in Hosein‐Sooklal and Kargol, 2002; Kargol, 2013).

A superposition of sine waves also formed the basis of a protocol introduced in Beattie et al. (2018). In this study, we measured *I*_Kr_ current in response to an 8 s optimized protocol, and used it to fit a four‐state Markov model (equivalent to a Hodgkin–Huxley model with two independent gates). The model was then used to predict the response to an AP waveform validation protocol (i.e., not used in the fitting data), and found to outperform models published in the literature which were fitted to conventional step protocols. Notably, this study performed a sinusoidal calibration protocol, and independent validation protocols in the form of (shortened versions of) conventional voltage‐clamp protocols and an APs clamp, all in the same cells. In a follow‐up publication (Clerx, Beattie, et al., 2019), we used this data set to compare four different fitting methods (each characterizing a common group of methods found in the literature) in terms of predictive power and robustness. These methods are summarized in Figure 5. In agreement with the theoretical studies discussed above, we found that whole‐trace fitting greatly outperformed fitting based on steady state and time‐constant approximation, and that the novel shortened protocol performed as well as its conventional counterparts.

Automated patch‐clamp platforms have recently become available, and offer a chance to perform experiments in much larger numbers than was possible before. A recent study by Lei, Clerx, Beattie, et al. (2019) adapted the information‐rich protocol work by Beattie et al. (2018) for these machines, replacing the sine waves by a staircase protocol (to overcome practical limitations) and creating 124 cell‐specific models in a single pass.

#### 3.1.5 Combining data sources

An advantage of optimization‐based fitting methods is that they provide a straightforward manner to incorporate data from different sources. For example, Vandenberg and Bezanilla (1991) and Irvine, Saleet Jafri, and Winslow (1999) combined measurements of whole‐cell current, single‐channel current, and gating‐current (the microscopic current generated by charge displaced in the channels' conformational changes) in a single model. More recently, measurements from voltage‐clamp fluorometry have been used in conjunction with electrophysiology data to calibrate and/or validate mathematical models of ion‐channel gating (Moreno, Zhu, Mangold, Chung, & Silva, 2019; Zaydman et al., 2014). While combining data this way has great potential, it can also lead to problems with finding a unique solution if the different data sets suggest a different “best” answer. This could happen if the model is known and accepted to be imperfect, in which case adjusting the weighting of the different data sets could allow a modeler to “tweak” the outcome, steering it toward a model assumed to be most useful in the expected context of use. But even if we do assume the model has the capability to fit all data sets at once, we might still expect difficulties from such a *multi‐objective* optimization problem, for example if each measurement was made on different cells or under slightly different conditions.

Finally, several authors have investigated fitting voltage‐sensitive ion‐channel models to data from sources other than whole‐cell current recordings. For example, single‐channel voltage‐clamp measurements (see below), but also single‐channel current clamp (Tveito, Lines, Edwards, & McCulloch, 2016). Several studies have investigated fitting to measurements of the AP and calcium transient (Bueno‐Orovio et al., 2008; Cairns, Fenton, & Cherry, 2017; Dokos & Lovell, 2004), which would allow kinetics to be determined for myocytes without using channel blockers or current‐separation protocols (see Section 4.3.2). Another approach to multichannel identification was introduced by Milescu, Yamanishi, Ptak, Mogri, and Smith (2008), who measured the AP in a cell and then *blocked the current of interest*, injected a simulated current using dynamic clamp, and adjusted the simulation parameters until the cell displayed its original behavior. This process can then be repeated by adding further blockers and models for subsequent currents.

### 3.2 Modulated voltage‐sensitivity

The next step up in complexity from purely voltage‐sensitive channels, is to study the situation where an ion current's voltage‐sensitive behavior is modulated by other factors, for example, temperature or binding of ligands. This comes into play in several ways in cardiac physiology, for example, when measurements are temperature‐adjusted to incorporate room‐temperature data or study fever and hypothermia, when studying the effects of mutations, drugs, or signaling (e.g., through channel phosphorylation), or when modeling currents like L‐type calcium which exhibit both voltage and Ca‐dependent kinetics.

Modifications to incorporate modulation can include changes to (a) the maximum conductance, for example, drug‐induced pore block (Mirams et al., 2011), mutations which reduce whole‐cell current (Sadrieh et al., 2014), or ion‐concentration dependence (Fink, Noble, Virag, Varro, & Giles, 2008); (b) kinetic parameters, for example, for temperature changes (Li et al., 2016), mutations (Clancy & Rudy, 2001), or again ion‐concentration dependence (Fink et al., 2008; Mazhari, Greenstein, Winslow, Marbán, & Nuss, 2001); or (c) the kinetic scheme itself, for example, to model the availability of drug‐receptor sites (Starmer, Grant, & Strauss, 1984) or to incorporate new “modes” created by mutations (Clancy & Rudy, 2002) or ligand‐binding (Bondarenko, 2014). For drug‐induced changes specifically, a useful overview can be found in Brennan, Fink, and Rodriguez (2009).

The simplest case, from a modeling point of view, is when channels can be split into an altered and an unaltered group, so that we simply perform inference twice to create two independent models whose combined behavior represents the situation of interest. For example, homozygous mutations in SCN5A (where a single gene encodes for the entire alpha subunit) have been modeled by simply replacing the entire *I*_Na_ model (Clancy & Rudy, 1999). Heterozygous mutations have been modeled by assuming a certain ratio between the two inherited types (Loewe et al., 2014), although often the heterozygous whole‐cell current is modeled as a single entity instead (Whittaker, Ni, Harchi, Hancox, & Zhang, 2017). For processes such as channel phosphorylation it is also common to include two independent channel models (O'Hara et al., 2011), but with a varying instead of a fixed ratio between the two.

An alternative representation of the same idea is to connect the two models in a single kinetic scheme, with (perhaps voltage‐insensitive) rate constants allowing the channel to switch from one mode to another. This representation is common for L‐type calcium models (Jafri, Rice, & Winslow, 1998; Mahajan et al., 2008) but has also been used to model drug effects (Brennan et al., 2009; Li et al., 2017).

For some modulating factors it can be more appropriate to keep the model structure fixed but modify the rate constants. This is a common strategy for temperature (Destexhe & Huguenard, 2000) but also for mutations (see Carbonell‐Pascual, Godoy, Ferrer, Romero, & Ferrero, 2016 for a discussion of modifying rates versus model structure). Recently, Lei, Clerx, Gavaghan, et al. (2019) measured and fitted *I*_Kr_‐kinetics independently at several temperatures, so that plots of the kinetic rates versus temperature could be made. Note that identifiability is crucial for such an exercise, as lack of identifiability could introduce meaningless changes in the obtained parameters (in this case, good identifiability properties had previously been shown [Lei, Clerx, Beattie, et al., 2019]). It may be possible to extrapolate this independent‐fits strategy to other areas, for example to see which rates are affected by a drug, and in this context shortened information‐rich protocols could be particularly useful. Finally, if an equation for the modulating effect on the rates is known, it may also be possible to vary both voltage and modulating factors during a single experiment, and fit a single model to the results.

### 3.3 Ligand‐gated channels

Purely ligand‐gated channels have not received as much attention in the cardiac literature as their voltage‐gated counterparts. A notable exception is the Ryanodine receptor (channel), which plays a crucial role in calcium handling and has been included in cardiac AP models since the work of Hilgemann and Noble (1987). In the neurological literature studies of ligand‐gated channels abound, with particular interest paid to the problem of fitting Markov models to single‐channel currents. For example, Colquhoun and Sigworth (1995), Horn and Lange (1983), and Bauer, Bowman, and Kenyon (1987) provide detailed early overviews of the statistical (maximum likelihood) methods involved, and Fredkin, Montal, and Rice (1985) derived an important result on rate identifiability. An overview of the early literature on this topic was compiled by Ball and Rice (1992), and a more recent assessment of maximum likelihood methods is given by Colquhoun, Hatton, and Hawkes (2003).

Several studies have addressed identifiability in this context, both analytically (Edeson, Ball, Yeo, Milne, & Davies, 1994) and using Bayesian sampling (MCMC) methods (Hines et al., 2014; Siekmann et al., 2012). The widespread use of statistical methods has also led naturally to likelihood‐based methods for model selection (Hodgson & Green, 1999; Horn & Vandenberg, 1984) and Bayesian analysis (Epstein, Calderhead, Girolami, & Sivilotti, 2016; Hodgson, 1999). In an interesting parallel with whole‐cell voltage‐clamp work, studies such as VanDongen (2004) have compared methods that pre‐analyze (“idealize”) the data before fitting, to methods that perform both steps simultaneously.

### 3.4 Pumps and transporters

Pumps and transporters perform key roles in cardiac myocytes, restoring the electrochemical gradients needed for passive transport through ion channels and refilling the sarcoplasmic reticulum after calcium release for contraction (Eisner, Caldwell, Kistamás, & Trafford, 2017). Despite this, the literature on modeling active transport in cardiomyocytes is sparse. For example, an analysis by Bueno‐Orovio, Sánchez, Pueyo, and Rodriguez (2014) found that Na/K pump formulations in AP were predominantly inherited from either DiFrancesco and Noble (1985) or Luo and Rudy (1994), while Niederer, Fink, Noble, and Smith (2009) showed that in this process the connection (and fit) to the source data was occasionally lost.

A very systematic approach to creating a model of the Na/K pump was taken by Smith and Crampin (2004). Starting from a detailed (but hard to parameterize) 15‐state kinetic scheme (the Post‐Albers model), they exploited the large differences in the model's (estimated) reaction rates to perform significant model reduction, leading to a simplified four‐state model with 14 parameters. Initial guesses for each parameter were made using a variety of published experimental results, after which optimization was used to fit the model to a published study of cycle rates versus voltage. Interestingly, some of the final rates shown in the article are equal to the initial values, which may indicate identifiability issues if the quality of fit was not found to vary when changing these parameters. A refinement of the model was made in Terkildsen, Crampin, and Smith (2007) using the same approach but fitting to an extended experimental data set. The same group then created a model of the SERCA pump, this time reducing a 12‐state kinetic scheme down to a 3‐state model, and again fitting to a combined data set from several sources (Tran, Smith, Loiselle, & Crampin, 2009). A recent publication by Pan, Gawthrop, Tran, Cursons, and Crampin (2019) introduces an extension of this approach, which uses *bond graphs* to derive reduced models that automatically obey laws of thermodynamics, this makes them more likely to give realistic predictions when extrapolating (see Section 2.2).

While current recordings from (human) cardiac pumps (especially at physiological temperatures) are rare, crystal structure data is available. A review by Gadsby (2009) uses these to argue that the principles behind active transport are more similar to passive ion‐channel transport than previously thought. If so, techniques used in the analysis of ligand‐gated channels may be applicable: while the current through a single pump is too small to be measured in isolation, studies have investigated extending single‐channel analysis methods to fitting to macroscopic data (Celentano & Hawkes, 2004; Milescu, Akk, & Sachs, 2005).

## 4 AP MODELS

In this section we discuss approaches to the calibration of (nonspatial) AP models, including advances in areas such as (a) representation of different species/cell types, (b) accounting for cell–cell variability, (c) reproducing the activity of individual cells, and (d) steps toward patient‐specific electrophysiology.

### 4.1 Conventional approaches

In the seminal work of Hodgkin and Huxley (1952), precise measurements of sodium and potassium ion‐channel kinetics were made in the squid giant nerve axon. These measurements were used to formulate differential equations representing ion channels with voltage‐dependent transition rates (see Section 3) which, when combined, were capable of accurately predicting the AP waveform of nerve impulses. Hutter and Noble (1960) and Noble (1965) showed through experimental and mathematical analysis that addition of an inwardly rectifying potassium current, *I*_K1_, and substantially slowed kinetics of the delayed rectifier potassium current, *I*_K_, allowed voltage waveforms very similar to those recorded in the Purkinje fibers of the heart to be reconstructed. However, application of voltage clamp and other techniques to cardiac tissue from the 1960s onward has revealed that a much larger array of ion channels, pumps and exchangers, and signaling processes are responsible for generating electrical activity in cardiac myocytes than are found in the simpler excitable cells of the squid giant axon (Bers, 2002; Roden, Balser, George Jr, & Anderson, 2002).

Early mathematical models of cardiac electrical activity included only a small number of ionic currents and clamped the values of intracellular ions (Beeler & Reuter, 1977; McAllister, Noble, & Tsien, 1975; Noble, 1962). The next cohort of models were updated to include newly discovered currents, pumps and exchangers, as well as intracellular processes (DiFrancesco & Noble, 1985; Hilgemann & Noble, 1987; Luo & Rudy, 1994). Given the dramatic increase in our knowledge and ability to characterize the rich ionic activity underlying cardiac electrophysiology during the latter part of the twentieth century, the approach of piecing together separate molecular‐scale models of ion currents, pumps, and exchangers has become increasingly prevalent, leading to many composite AP models which comprise components from different cell types and even species (Niederer et al., 2009). When developing AP models, once separate ion‐channel models are calibrated and pieced together, the contribution of these interacting cellular components has to be tuned via the maximal conductance to reproduce voltage‐clamp experiments, physiological AP waveforms, as well as ideally other cellular‐level emergent properties such as rate‐adaptation and recovery of excitability. As conductance and permeability parameters (which describe the density of ionic currents) represent properties of the cell, this process is typically treated independently from that of estimating kinetic parameters, which are usually treated as fixed properties of ion channels.

We also note that since at least the Hilgemann and Noble (1987) model, most biophysically detailed AP models have included not just currents passing through the outer membrane but also Ca^2+^‐handling processes relating to subcellular structures such as the sarcoplasmic reticulum and T‐tubules which also affect the AP and require calibration. While we believe that many of the same principles apply in calibrating the Ca^2+^ subsystem components as those on which we focus for outer membrane electrophysiology, it should also be noted that there are some unique challenges associated with calibrating models of Ca‐handling and Ca‐dependent processes.

Calcium is involved in multiple, complex feed‐forward, and feedback interactions which involve numerous ion currents, proteins, and interacting pathways (Winslow, Walker, & Greenstein, 2016). Not surprisingly, there are controversies in the proposed mechanisms by which Ca^2+^ regulates different cardiac myocyte processes, which arise from differences in measurement methodologies between labs, the fact that calcium is buffered by various reactions, inability to measure certain quantities directly, and incomplete understanding of the underlying biophysics (Bers, 2008; Rice & Jafri, 2001; Winslow et al., 2016). An early example of a challenge encountered in calibrating Ca‐handling in AP models is that accurate modeling of calcium‐induced calcium release (specifically the graded release of Ca^2+^ from the sarcoplasmic reticulum and high gain) requires that multiple intracellular domains exist with differing local concentrations of calcium, rather than a “common‐pool” construction (in which Ca^2+^ influx across the sarcolemma and release from the sarcoplasmic reticulum is into the same compartment) as is often used (Rice & Jafri, 2001). The relative sizes, locations, and fluxes between domains/compartments thus require calibration, based on intracellular data which are difficult to measure and may not be available, making this a real challenge.

In terms of electrophysiology, Ca‐dependent inactivation is thought to be the predominant mechanism of L‐type calcium channel current inactivation (Linz & Meyer, 1998), and thus a model's Ca^2+^ dynamics can critically influence the AP morphology and available mechanisms of arrhythmogenesis. Characterizing quantitatively the fraction and rate of L‐type calcium channel current inactivation with calcium concentration is difficult, as intracellular calcium cannot be easily clamped to a constant value (ten Tusscher & Panfilov, 2006). It is finally worth mentioning that Ca^2+^ modulates 7cardiac myocyte behavior in ways which extend beyond electrophysiology which may be of interest to modelers, as Ca^2+^ is critically involved in cardiac contraction, energy production, signaling, and transcription. Calibration of other Ca‐dependent processes must therefore ensure that Ca^2+^ dynamics allow simulation of these additional phenomena while not interfering with excitability (and contractility). While a full discussion of efforts to quantitatively model Ca^2+^ regulation of cardiac myocytes is beyond the scope of this review, we refer the interested reader instead to a recent review (Winslow et al., 2016).

**Figure 6:**
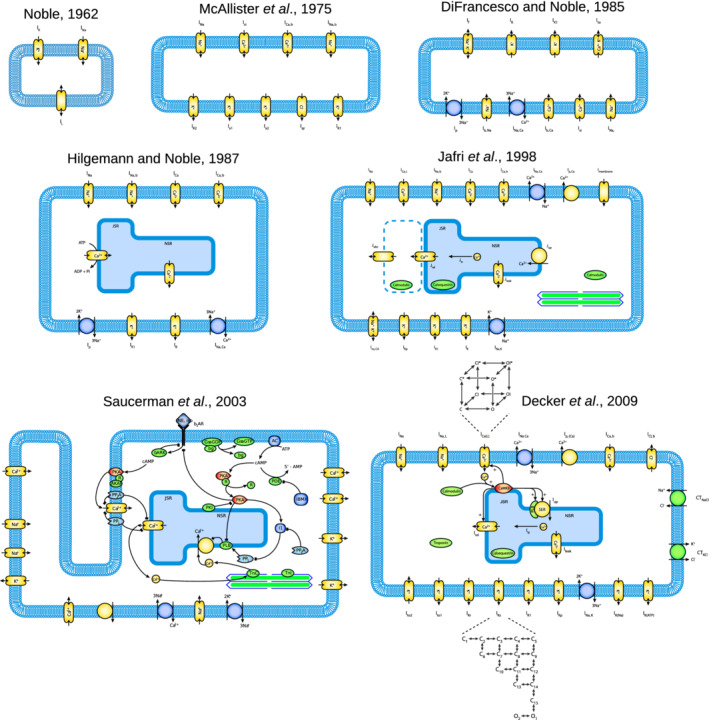
The evolving complexity of cardiac AP models. Selected action potential model schematics are presented in chronological order, including the models of Noble (1962) (the first model of the cardiac AP), McAllister et al. (1975) (which added more heart‐specific currents), DiFrancesco and Noble (1985) (which added ion pumps and exchangers), Hilgemann and Noble (1987) (which represented a significant advance in modeling calcium dynamics), Jafri et al. (1998) (which added calcium dynamics to the commonly used Luo & Rudy, 1994 model), Saucerman, Brunton, Michailova, and McCulloch (2003) (which modeled *β*‐adrenergic signaling), and Decker, Heijman, Silva, Hund, and Rudy (2009) (which included Markov formulations of both *I*_CaL_ and *I*_Ks_). All cell‐model schematics are available from (https://models.cellml.org/cellml) under a CC‐BY license. AP, action potential

**Figure 7:**
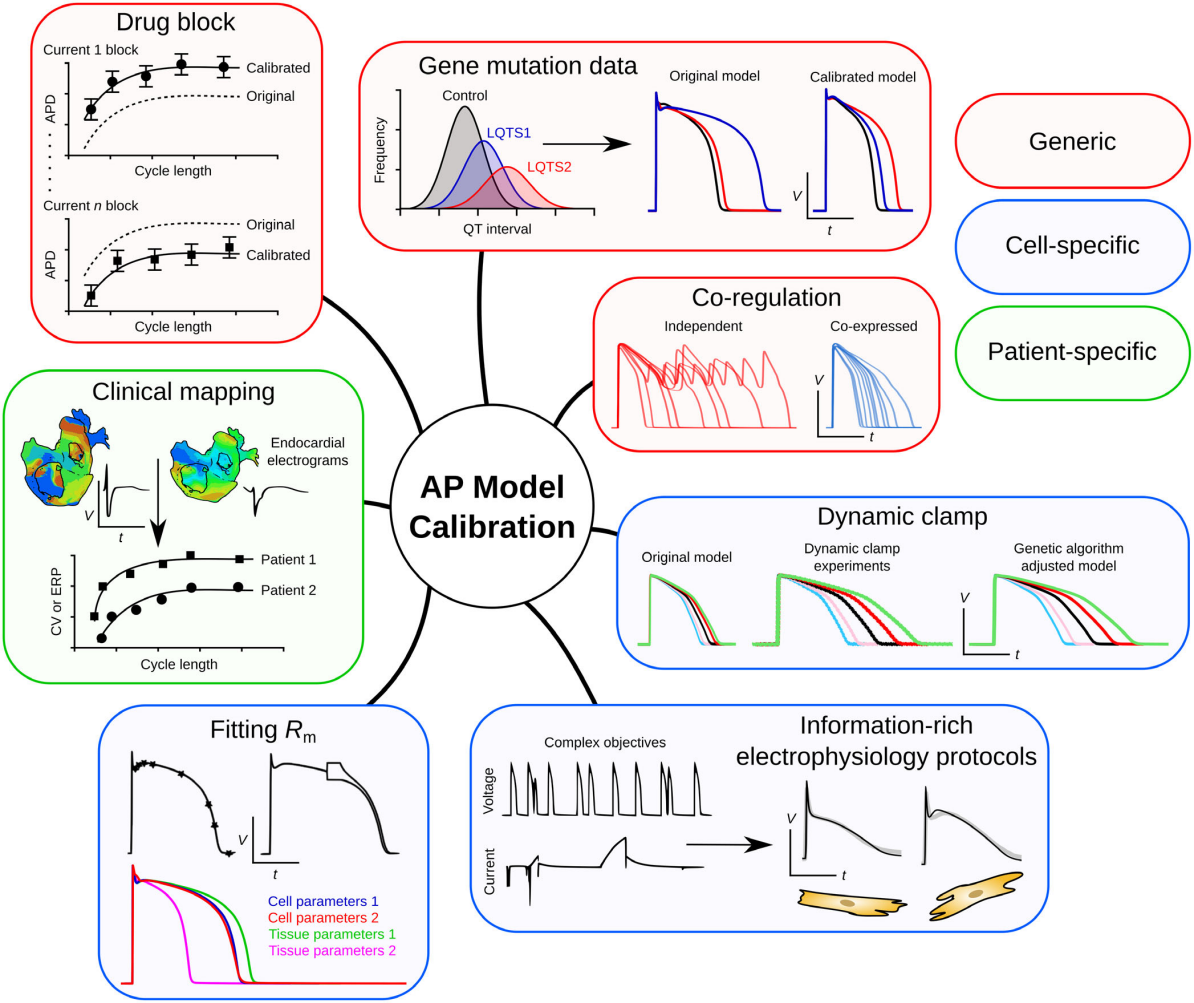
A summary of some of the methods outlined in this review used to calibrate existing models of the action potential (AP). Methods are colored according to whether they represent a generic approach (red), cell‐specific approach (blue), or patient‐specific approach (green). Generic approaches shown include recalibrating ionic current balance using clinical gene mutation data (Mann et al., 2016), by accounting for co‐regulation of ion‐channel expression (Ballouz et al., 2019), and based on drug block measurements (Dutta et al., 2017). Cell‐specific approaches illustrated include adding the membrane resistance, *R*_m_, to the function objective (Kaur, Nygren, & Vigmond, 2014), using information‐rich voltage and current clamp electrophysiology protocols to identify cell‐specific conductances (Groenendaal et al., 2015), and using genetic algorithms to adjust ionic conductances based on dynamic clamp experiments (Devenyi et al., 2017). The patient‐specific approach shown involves using endocardial electrogram clinical mapping data to tailor simplified models of human atrial electrophysiology to individual patients (Corrado et al., 2017, 2018)

A selection of AP model schematics (taken from https://models.cellml.org/cellml) illustrate the evolving complexity of cardiac cellular electrophysiology models in Figure 6. An illustrated summary of some of the methods used to calibrate AP models discussed in Section 4 is given in Figure 7.

#### 4.1.1 Calibration of ionic current densities

The traditional approach to calibrating ionic current densities during AP model development is to measure *I*–*V* curves in (major) currents of interest, and to derive a maximal conductance for each of these based on fitted peaks (assuming individual current measurements are specific and not polluted by other currents). The sodium current is often treated independently, by choosing its conductance to be a value which gives an appropriate maximum upstroke velocity. The conductances of remaining ionic currents (which have not been measured directly) may be inherited from earlier models (of a different species/cell type), based on literature values, or tuned to reproduce a certain physiological behavior or to minimize some aspect of model discrepancy, which may involve optimization (Fabbri, Fantini, Wilders, & Severi, 2017).

Optimization approaches are not common, and calibration of ionic current densities is often done manually using subjective validation criteria. This can lead to an inadequate exploration of the parameter space, resulting in suboptimal fits (Krogh‐Madsen, Sobie, & Christini, 2016). A more rigorous approach to model calibration is to treat AP model development as a parameter estimation problem in which voltage‐clamp traces, biomarkers or whole AP time series are used to fit the conductance parameter values (Hafner, Borchard, Richter, & Neugebauer, 1981). Application of this method often reveals parameter identifiability issues (Dokos & Lovell, 2004; Johnstone, Chang, et al., 2016). In part to address this, a range of additional sources of information have been used in the objective function, such as a more complex stimulus protocol (Dokos & Lovell, 2004; Groenendaal et al., 2015), intracellular Ca^2+^ transient morphology (Passini et al., 2016; Rees et al., 2018), and action potential duration (APD) restitution (Cairns et al., 2017; Syed, Vigmond, Nattel, & Leon, 2005), in order to increase parameter identifiability. A recent attempt has been made to separate more clearly the processes of model calibration and validation when using an optimization algorithm to fit current densities in a human ventricular model by Tomek et al. (2019).

#### 4.1.2 Accounting for experimental conditions

Another way to distinguish between parameter sets which yield similar outputs (Sarkar & Sobie, 2010; Zaniboni, Riva, Cacciani, & Groppi, 2010) can be to account for conditions experienced by a real cell during the experiment, such as electrotonic coupling (depending on the preparation) or compensatory currents. For example, Al Abed, Guo, Lovell, and Dokos (2013) presented a tissue‐based optimization process in which a phenomenological model was fitted to heterogeneous APs from cardiac myocytes under electrotonic load (for more on reduced/phenomenological models see Section 4.4). The advantage of such an approach stems from the fact that implementation of parameters from single‐cell ionic models into spatially extended domains might not be able to reproduce expected properties such as an appropriate conduction velocity (CV) under physiological intercellular coupling (Elshrif & Cherry, 2014), or spontaneous pacemaking activity from the center of the sinoatrial node (Garny, Kohl, Hunter, Boyett, & Noble, 2003). An alternative way of incorporating information pertaining to tissue‐level electrophysiology was presented by Kaur et al. (2014), who demonstrated improved parameter estimation and reduced variability in ionic conductances of mathematical AP models when including the membrane resistance, *R*_m_, at various points along the AP in the function objective, which provides information about how sensitive the AP waveform is to perturbations in current (or current flow along adjacent myocytes in tissue).

Colman, Saxena, Kettlewell, and Workman (2018) built “lab‐specific” models of the human atrial AP *during patch clamping*, based on currents measured in the Workman laboratory. The developed models (a) included a compensatory hyperpolarizing current, *I*_hyp_, used in human atrial patch‐clamp electrophysiology experiments to stabilize the resting membrane potential (Workman et al., 2006), and (b) lacked *I*_Kr_ and *I*_Ks_, which may be disrupted by the cell isolation process (Yue, Feng, Li, & Nattel, 1996). The AP morphology between “isolated” and “intact” variants of the models differed significantly, where “isolated” cell APs showed a triangular AP morphology with slow repolarization tail, similar to that observed in the Workman lab, whereas “intact” cell APs exhibited more of a “spike‐and‐dome” morphology, consistent with some literature models (Courtemanche, Ramirez, & Nattel, 1998). This highlights the importance of accounting fully for the experimental conditions in model calibration and validation, as inaccurate ionic current kinetics/conductances may be used to account for significant model discrepancy arising from missing components such as *I*_hyp_.

#### 4.1.3 Recalibration in a new context of use

Due to a frequent lack of adequate validation of AP models, as well as the often manual approach of calibrating the numerous ion‐channel conductances in a model, “out of the box” models can make spurious predictions in new situations (Elshrif & Cherry, 2014; Noble et al., 2012). Furthermore, different models of the same species often produce divergent results when predicting the response to the same perturbation (Mann et al., 2016; Mirams et al., 2014). One approach to circumvent this problem to some degree is to “recalibrate” the ionic current balance in existing AP models, which can be achieved through global optimization to additional data such as the response to drug block or genetic mutation. For example, in Dutta et al. (2017), the maximal conductances of *I*_Kr_, *I*_Ks_, *I*_K1_, *I*_NaL_, and *I*_CaL_ in the O'Hara et al. (2011) model were adjusted to better reproduce APD prolongation under drug block conditions, that is, E‐4031 (*I*_Kr_ block), HMR‐1556 (*I*_Ks_ block), mexiletine (*I*_NaL_ block), nisoldipine (*I*_CaL_ block), and BaCl_2_ (*I*_K1_ block). This vastly improved overall concordance of the model with experimental results under a range of drug block conditions. This is important considering the context of use; the improved model is intended to provide a foundation for using in silico models for the regulatory assessment of torsades de pointes (TdP) risk under the comprehensive in vitro proarrhythmia assay (CiPA) paradigm (Li et al., 2019).

A similar approach to recalibrating ionic current balance is that of Mann et al. (2016), who optimized not just one but three leading human ventricle models to clinical QT prolongation data associated with long QT syndromes (LQTS). Where previously the models produced divergent, unrealistic predictions of the response to channel block, after global optimization the models showed convergent behavior. It was shown subsequently in Krogh‐Madsen, Jacobson, Ortega, and Christini (2017) that placing physiologically based bounds on intracellular ionic concentrations, in addition to optimization to LQTS data, improved prediction of TdP risk using the data sets of Lancaster and Sobie (2016) and Mirams et al. (2011). The specific example of verapamil, which blocks *I*_Kr_ but does not cause TdP and can thus be difficult to classify (Britton, Abi‐Gerges, et al., 2017; Dutta et al., 2017; Mirams et al., 2011), was highlighted. Where the uncalibrated model produced a significant APD prolongation under application of verapamil, the multivariable optimized ORd model produced only a minor prolongation, consistent with lack of QT prolongation (Redfern et al., 2003). These studies highlight the potential of such global optimization methods to improve the accuracy and credibility of in silico cardiotoxicity prediction, while also highlighting areas which require further attention/refinement in the models.

#### 4.1.4 Translation across cell types

Much of biomedical research is based on extrapolation of findings from one cell type or species to another, due to a range of financial, ethical, and technical considerations that may be associated with performing experiments in the desired cell type (typically an adult, human cardiomyocyte). Approaches must therefore be developed which can improve translation of findings between cell types and species, as well as subpopulations and disease states, to enable interpretation and quantitative analysis in the context of the desired cell type. Calibration of mathematical models to reproduce cross‐cell type differences in cardiac electrophysiology is one relatively established approach with many applications, such as translation of findings from transgenic mouse models to human physiology, and improvement of drug cardiotoxicity prediction pipelines, in which drug effects are now being measured in hiPSC‐CMs with the aim of predicting effects in humans (Colatsky et al., 2016). We here differentiate model calibration pertaining to cross‐cell type modeling into two categories: (a) calibrating existing models to generate models of new cell types, and (b) using mathematical and modeling methods to predict the behavior of one cell type based on measurements made in a different cell type.

A common approach to deriving models of new cell types from existing models is to simply tune model conductances based on measured differences in current density, mRNA expression, or other relevant properties, between cell types, thus requiring no parameter estimation. This general approach of tuning (predominantly) maximal conductances in AP models has been used to generate models of: regional differences within the heart (Zhang et al., 2000); male and female differences (Yang & Clancy, 2012); the aging sinoatrial node (Behar & Yaniv, 2017); and pathological conditions such as atrial fibrillation (AF; Colman et al., 2013); and heart failure (Ponnaluri et al., 2016), to name just a few.

Use of ion‐channel mRNA expression to calibrate models of cardiac electrophysiology is controversial, as the relationships between mRNA and protein, and protein and function, are not necessarily linear (Boyett, 2009). Nonetheless, it was shown by Chandler et al. (2009) that simply scaling maximal conductances in the Courtemanche et al. (1998) human atrial model based on mRNA expression between the right atrium and sinoatrial node was able to produce a nodal AP morphology and pacemaking. Furthermore, some ventricular electrophysiology models use mRNA expression scalings as the basis for transmural cell types (O'Hara et al., 2011).

As for the second problem, that of predicting the behavior in one cell type based on observations made in a different cell type, Ahrens‐Nicklas and Christini (2009) presented a hybrid computational–experimental approach to translating interspecies differences in cardiac electrophysiology. The cell type transforming clamp (CTC) is a variant of the dynamic clamp technique which uses a compensatory current (based on the difference in net membrane current between two mathematical models—one a model of the same species as the target cell, and the other a model of the desired species) calculated in real time to convert the macroscopic electrical behavior of an isolated cell into that of a different cell type. In this case, the technique was applied to murine ventricular myocytes in order to “anthropomorphise” the AP to behave like human ventricular myocytes, and required injection of a large, sustained inward current. The effectiveness of CTC relies on a small model mismatch between the target‐canceling mathematical AP model output and the target cell AP. It was subsequently shown that the CTC technique can be improved by using a genetic algorithm to rapidly tune the target‐canceling model in real time to better reproduce the AP of the target myocyte, thus reducing model mismatch and improving CTC performance (Bot, Kherlopian, Ortega, Christini, & Krogh‐Madsen, 2012).

A method for quantitatively predicting the effects of drugs in one cell type based on measurements made in another cell type, was presented in Gong and Sobie (2018), using mathematical modeling and statistical analyses. A cross‐cell type multivariable regression model was constructed between hiPSCs and human ventricular cardiomyocytes, enabling prediction of the response of a mature ventricular cardiomyocyte model to ionic conductance perturbations from measurements of multiple biomarkers (pertaining to voltage and calcium) made in hiPSCs. Although the study was conducted using synthetic data, such approaches may be of great value in the drug development process. An alternative method, based on identifying parameter vectors which can predict the effects of drugs in mature cardiomyocytes, based on measurements made in “immature” hiPSCs, can be found in Tveito et al. (2018) and Jæger, Charwat, et al. (2019).

### 4.2 Variability approaches

The majority of models of cellular cardiac electrophysiology utilize “population‐averaged” data as the basis for model components, which do not account for inter‐ and intra‐sample variability. This approach can therefore fail to represent any specific individual or emergent property well (Golowasch, Goldman, Abbott, & Marder, 2002; Marder & Goaillard, 2006; Pathmanathan et al., 2015). In light of this deficiency, different methods harnessing the power of modern computers have begun to emerge which account for variability in cardiac electrophysiology—see Ni, Morotti, and Grandi (2018) for a recent review—some of which will be discussed in this section.

#### 4.2.1 Representing variability with parameter samples

One way to represent interindividual variability is by assigning different parameter sets within the same model to represent different individual cells, animals or patients. The “population of models” approach does this by varying conductances (and sometimes a few important kinetic parameters) to create many parameter variants, and then discards those that are inconsistent with experimentally measured ranges of cellular‐level biomarkers. It was introduced in a study by Britton et al. (2013), in which 10,000 parameterizations of rabbit Purkinje fibers were generated randomly, and 213 parameter sets with outputs within the ranges of the marginal distributions of several biomarkers were retained. These experimentally calibrated parameter sets, which showed parameter variations of up to ±100% for some conductances, were then used to predict the APD prolongation associated with four concentrations of the *I*_Kr_ blocker dofetilide.

Subsequent applications of this population‐of‐models approach are reviewed in Muszkiewicz et al. (2016). More recent applications include a proarrhythmic drug risk marker based on the approach (Passini et al., 2017); calibration of parameters based on recordings from human ventricular trabeculae (Britton, Bueno‐Orovio, et al., 2017); evaluation of antiarrhythmic effects of mexiletine and ranolazine on the LQTS3 phenotype in hiPSC‐CMs (Paci, Passini, Severi, Hyttinen, & Rodriguez, 2017); and refinement of the population through including optical calcium signal biomarkers as extra constraints (Paci et al., 2019). Drovandi et al. (2016) compared three sampling methods and presented an improved approach for sampling from high‐dimensional parameter spaces, an important step in the initial calibration of a “population of models.”

Calibration of “populations of models” involves discarding parameter sets which exhibit properties which lie outside experimentally measured ranges. The steps taken are the same as (nonsequential) approximate Bayesian computation (ABC), assuming a uniform prior, or a single wave of the “history matching” technique (Andrianakis et al., 2017; Vernon, Goldstein, & Bower, 2014). The subsequent interpretations differ, as a history matching approach describes the remaining region as “not implausible” or “not ruled out yet” parameter space, rather than a plausible explanation of variability in the data.

Bayesian history matching was applied to the calibration of maximal conductances of ion channels and exchangers in two human atrial AP models against experimentally measured AP biomarkers in Coveney and Clayton (2018). In that study, and indeed typically, the use of history matching is accelerated with Gaussian process emulators (fast‐running surrogate models) using a small number of model runs (∼10^2^ runs) to train an emulator, which could be run many times (>10^6^) at low computational cost with different sets of parameters. By iteratively repeating the process of comparing emulator outputs with experimental biomarkers, the region of not implausible parameter space is reduced at each wave. Both the Courtemanche et al. (1998) and Maleckar, Greenstein, Giles, and Trayanova (2009) human atrial AP models could be calibrated successfully to experimental datasets (except for the AP amplitude biomarker which could not be reproduced by Maleckar et al., 2009 model due to model mismatch), resulting in sets of parameters that could be sampled to produce variable APs.

A range/distance‐based calibration prevents unphysiological models from being accepted into a population, but does not guarantee that the distributions of biomarkers in the experimental data are well matched by the parameter sets. Lawson et al. (2018) demonstrated an approach for calibrating “populations of models” to distributions of outcomes, as a way of reducing this bias. While the method was mostly able to capture the variability inherent in the experimental data, varying conductances alone was insufficient to simultaneously match distributions of AP amplitude and *dV*/*dt*_max_, as models showed a strong correlation between these two biomarkers which was not present in the data. Introducing a parameter which scaled the time constant of sodium channel inactivation was shown to allow greater decoupling of AP amplitude and upstroke velocity, although the correlation between these two biomarkers remained stronger than in the data. This inconsistency reveals that assumptions about the sources of variability in the data were too restrictive when considering conductances only, and/or there is discrepancy in the model's current kinetics.

#### 4.2.2 Co‐regulation

An important area for refinement of population modeling approaches is that co‐expression of certain ion channels may occur in reality (Banyasz, Horvath, Jian, Izu, & Chen‐Izu, 2011; Eichel et al., 2019; Rees et al., 2018), which places additional constraints on model parameters. In Ballouz et al. (2019), meta‐analyses of public RNA‐seq datasets and extraction of mRNA from hiPSC‐CMs revealed a strong correlation between expression of *CACNA1c* (*I*_CaL_) and *KCNH2* (*I*_Kr_). In silico modeling of populations of human ventricular cells showed that co‐expression of *I*_CaL_ and *I*_Kr_ limited the variability in APD and reduced susceptibility to EADs, an important marker of proarrhythmia.

Relevant in this regard is the concept of “good enough solutions,” which is that real biological systems are sufficiently complex that countless possible combinations of the constituent elements (e.g., ion‐channel gene expression) can produce equivalent outputs, or rather, a “good enough solution” to accomplish normal biological function (Weiss et al., 2012). However, in the face of environmental stresses, the “good enough solutions” of certain individuals respond more favorably than others, which may explain variability in individual susceptibility to disease and adverse drug effects. It has been hypothesized that this serves an evolutionary advantage, allowing populations to adapt and survive in the face of new environmental stresses (Marder & Goaillard, 2006).

Rees et al. (2018) constructed a population of “good enough solutions” of mouse ventricular cells, in which conductances were constrained only by Ca^2+^ sensing, that is, biomarkers relating to intracellular calcium transients (and sodium), with no constraints on the AP waveform. This strategy revealed that AP waveforms showing large variability could produce roughly the same Ca^2+^ transient. However, a tight three‐way compensation between *I*_CaL_ and *I*_to,*f*_ + *I*_Kur_ was also observed, suggesting that Ca^2+^ sensing alone may be sufficient to regulate ionic conductances. Voltage‐clamp measurements of these currents in nine isogenic strains from the Hybrid Mouse Diversity Panel were used subsequently to validate the computational “good enough solutions,” where good agreement regarding co‐expression of these currents was seen. The study thus proposes a mechanism by which inward Ca^2+^ and outward K^+^ currents compensate each other to generate a normal Ca^2+^ transient in ventricular myocyte populations, with no requirements for voltage sensing. This “Ca^2+^‐centric” view of the constraints on ion‐channel expression has implications for population‐based modeling in cardiac electrophysiology, in which parameter searches tend to be “AP‐centric,” which may give rise to unphysiological Ca^2+^ transients in spite of physiological AP waveforms. Furthermore, the large variability in AP waveforms reveals that ion current conductances likely cannot be identified uniquely from the intracellular calcium transient alone.

#### 4.2.3 Mixed effects and hierarchical models

To date, little has been done in the way of UQ for cellular models of cardiac electrophysiology (Mirams et al., 2016). In Pathmanathan et al. (2015), uncertainties in parameters describing steady‐state inactivation of the sodium current, based on voltage‐clamp data variability from a population of canine ventricular myocytes, were propagated through forward runs of a canine ventricular AP model. An “individual‐based” statistical methodology, nonlinear mixed effects modeling, was shown to offer advantages over the conventional “population‐averaged” approach, which failed to capture the characteristics of any individual cell, by consistently underestimating the slope of activation. This “failure of averaging,” well known in neuronal modeling (Golowasch et al., 2002; Marder & Goaillard, 2006), has implications for cardiac modeling—use of “population‐averaged” ion current data is ubiquitous in ion‐channel model calibration (most often because this is the only form in which the data are available). At the single‐cell level, forward uncertainty propagation revealed that the model was not robust to the underlying variability—a large distribution of outputs on the maximum upstroke velocity was observed, and the APD could not be computed for a relatively large region of parameter space due to repolarization failure, which is clearly unphysiological behavior. In contrast, tissue‐level spiral wave dynamics were highly robust to the underlying variability, showing almost no differences in spiral wave core trajectories for parameter sets covering a large region of the two‐dimensional parameter space. Pathmanathan et al. suggested that this may represent a kind of *emergent robustness*, that is an inherent robustness to natural variability in function across multiple scales in systems biology.

An alternative way of dealing with variability within a hierarchical Bayesian framework was presented in Johnstone, Bardenet, et al. (2016). Synthetic AP traces were generated using different sets of parameters, and were then “forgotten” and their probability “posterior” distributions re‐inferred using MCMC methods. In other words, this approach entails finding all parameter sets which allow the model to be fit to the data, while assigning these sets of parameter values different probability densities according to how good a fit they provide. It was found that “correct” distributions for most ionic current densities could be found for each AP trace, as well as an approximation to the higher‐level distribution from which these parameter values were sampled. In addition, increasing the number of datasets being fit to improved posterior predictive distributions by increasing overall information content, albeit at the expense of computational speed. However, some level of parameter unidentifiability existed among some of the current densities.

### 4.3 Cell‐specific models

As discussed in the previous section, cardiac electrophysiology shows a large degree of inter‐ and intra‐cell variability. Mathematical models of cellular cardiac electrophysiology, on the other hand, typically represent non‐specific, aggregates of multiple ionic currents measured under varying conditions in different labs, cell types and even species (Niederer et al., 2009). An alternative to modeling this variability with samples of parameters is to calibrate models of the AP to reproduce activity from individual cells, thus providing a higher degree of quantitative predictive accuracy than is possible with generic, nonspecific models. In this case, one would study variability by making large numbers of cell‐specific models and examining how their parameters vary (Ni et al., 2018), automatically capturing any co‐regulation mentioned above.

#### 4.3.1 Identification of cell‐specific conductances

Given the huge number of parameters in most modern models of cardiac electrophysiology, attempts to tailor models to individual cells are typically restricted to identifying cell‐specific conductances (or current densities for pumps/exchangers). For example, Davies et al. (2012) calibrated the Hund and Rudy (2004) canine ventricular AP model to AP traces from 19 different dogs by adjusting the conductances of ionic currents. This formed an ensemble of model variants that better reproduced the AP morphology and dofetilide‐ and diltiazem‐induced APD_90_ changes than the uncalibrated model. With regards to the identifiability of parameters, Sarkar and Sobie (2010) demonstrated that some parameters of cardiac electrophysiology models can be identified using inverse regression (i.e., predicting an input matrix based on an output matrix), if the number of linearly independent outputs equals the number of inputs. Using the 16 best outputs from a model, it was shown that 12 out of 16 parameters could be inverted with *R* ^2^ > 0.77 in the ten Tusscher and Panfilov (2006) model.

Genetic algorithms are inspired by evolutionary biology. They are initialized by randomly varying maximal conductances and important kinetic parameters, from which the population evolves iteratively toward better solutions with each generation, through the processes of *selection* of good parameter sets, *crossover*, and *mutation* (Holland, 1992). The feasibility of fitting an existing mathematical model to an arbitrary AP waveform using a genetic algorithm was first demonstrated by Syed et al. (2005), who showed that both the Nygren et al. (1998) and Courtemanche et al. (1998) models of the human atrial AP could be fitted to experimentally recorded APs over several stimulation frequencies.

Groenendaal et al. (2015) demonstrated the feasibility of generating cell‐specific cardiac electrophysiology models using genetic algorithms, while investigating the issue of parameter identifiability. It was shown, using synthetic data, that a single AP was not sufficient to constrain the parameters of the Faber and Rudy (2000) guinea pig AP model, but that stochastic aperiodic pacing helped to improve identifiability of maximal conductances. Combining this with an optimized voltage‐clamp protocol which consisted of several steps, each one aiming to maximize the sensitivity of a single current, gave the best estimate of Faber and Rudy (2000) model parameters. This “information‐rich” protocol was subsequently tested in four guinea pig left ventricular myocytes (by switching from current clamp to voltage‐clamp mode). Mathematical models of the guinea pig AP calibrated to the experimental data were shown to predict the response to a novel stochastic simulation sequence far better than the original “uncalibrated” model.

Johnstone, Chang, et al. (2016) elucidated the requirements for identifiability of maximal conductances in numerous AP models ranging in complexity from the Hodgkin and Huxley (1952) model which has just three conductance parameters to be inferred, to the Davies et al. (2012) model which has 14. Using synthetic data, it was shown that for some of the more complex models, there were no simple pacing protocols which could identify all conductances, highlighting parameter identifiability issues. Similar conclusions were reached by Jæger, Wall, and Tveito (2019), who showed that a random stimulation protocol similar to that employed in Groenendaal et al. (2015) greatly increased the identifiability of conductances in three cardiac models (but did not allow complete identifiability in any model).

The genetic algorithm approach was combined with dynamic clamp experiments in Devenyi et al. (2017), in which a model of the guinea pig AP was calibrated to experimental data using simulated dynamic clamp experiments (where injected current does not have ion selectivity). Current block using the simulated dynamic clamp technique produced significantly different results from those obtained in real guinea pig left ventricular myocytes, tending to underestimate the degree of APD prolongation/shortening. The model was subsequently calibrated using a genetic algorithm applied to the input and output from the dynamic clamp experiments. The adjusted model had significantly reduced *I*_Ks_, increased *I*_Kr_, and reduced *I*_CaL_. Independent validation experiments in which *I*_Ks_ was blocked with chromanol 293B showed a modest APD prolongation, which was predicted by the adjusted model, but not the uncalibrated model.

A simple approach for tailoring mathematical models of hiPSC‐CMs for improved prediction of drug‐induced changes to the AP was outlined in Lei et al. (2017). It was shown that measurements of inward and outward currents could be used to reparameterize conductances in mathematical models of iPSC‐CMs, creating tailored models which had improved predictive power compared to the untailored mathematical model of Paci, Hyttinen, Aalto‐Setälä, and Severi (2013). The experiments also uncovered potential differences in Cor.4U and iCell iPSC‐CM cell lines, which highlights the need for cell‐line or even cell‐specific models of iPSC‐CMs.

#### 4.3.2 Beyond cell‐specific conductances

Some studies have attempted to address whether it is possible to estimate kinetic parameters of multiple ionic models simultaneously on a cell‐specific basis, using measurements which are available to experimenters. For example, Dokos and Lovell (2004) attempted to estimate 63 parameters of the Beeler and Reuter (1977) model. Calibrating to an AP waveform and a series of perturbed waveforms generated by current injections allowed reconstruction of ionic current profiles which are “hidden” (from the point of the experimenter), although fitted parameters still deviated by a mean value of 10.5% from published values, despite a high degree of prior information being assumed for each parameter.

Shotwell and Gray (2016) used some of the conventional optimal experimental design criteria (see Section 2.6.3) to assemble a set of experiments that can inform the 13 parameters of a modified Hodgkin and Huxley (1952) model (including a diffusion coefficient and propagation experiments). They showed that different experimental design criteria result in different weights being assigned to different experiments. Phenomenological models (see Section 4.4 for more on reduced/phenomenological models) may offer advantages in this respect, as parameters can be related to electrophysiological quantities, which makes initial parameterization easier (Bueno‐Orovio et al., 2008; Oliver & Krassowska, 2005).

In Cairns et al. (2017), genetic algorithms were used to estimate all parameters in two phenomenological models when fitting to arbitrary AP waveforms. It was found that good parameterizations for such models could be found in as little as 30–40 s, which were able to fit APs at cycle lengths used in the fitting process as well as those that were not. Furthermore, good agreement was achieved when fitting the phenomenological models to APs recorded experimentally using micro‐electrodes. However, significant variability was observed in the parameter values obtained, highlighting issues of parameter identifiability.

### 4.4 Reduced, phenomenological, and parsimonious models

As outlined in Section 4.3, current generation models of cardiac electrophysiology typically contain parameters relating to numerous interacting ion current and calcium handling processes, which may result in parameter unidentifiability (Johnstone, Chang, et al., 2016), and dissimilarity in predictions, even between models which purport to represent the same biological system (Elshrif & Cherry, 2014; Mirams et al., 2014; Wilhelms et al., 2013). This means it has become increasingly difficult to understand the relationship between model outputs and model parameters (Jæger et al., 2019), suggesting a need for more frugal, reduced‐complexity models of the cardiac AP.

One historic motivation for creating reduced models is that complex models with many ODEs may not be suitable for simulations of cardiac tissue electrophysiology in spatially extended domains which are typically demanding in terms of computational resources (Aliev & Panfilov, 1996; Fenton & Karma, 1998; Mitchell & Schaeffer, 2003), although this is less of a barrier today. Reduced models exchange biophysical detail for minimal phenomenological representations of a system which are able to reproduce emergent phenomena such as rate dependence of the AP and spiral waves in tissue. Additional advantages of such models are that parameters tend to be easily identifiable, as each process (e.g., depolarization, repolarization rate) may be controlled by as little as one parameter, as opposed to complex models, in which many combinations of the constituent ionic currents can give rise to the same AP waveform (Sarkar & Sobie, 2010; Zaniboni et al., 2010).

#### 4.4.1 Model reduction

Through sensitivity analysis and attempts to uniquely identify parameters, it is often found that there is some model redundancy, that is, there are parameters/currents which have little to no effect on the model output in most contexts, or can co‐vary with other parameters such that the same output could occur in different ways. Several authors have exploited this redundancy to create “reduced” versions of more complex models, which may accurately reproduce the behavior of the original model at a fraction of the computational cost. For example, 2‐ and 3‐variable reductions of the 14‐variable Rasmusson, Clark, Giles, Shibata, and Campbell (1990) pacemaking model were presented by Herrera‐Valdez and Lega (2011). More recently, Lombardo and Rappel (2017) used the manifold boundary approximation method to systematically reduce the Koivumäki, Korhonen, and Tavi (2011) human atrial model, which originally contained 42 variables and 37 parameters, to a model with only 11 variables and 5 parameters. The reduced model could reproduce the AP morphology and restitution of the original model, while decreasing simulation time more than threefold. Furthermore, each variable and parameter could be related to electrophysiological quantities.

#### 4.4.2 Phenomenological or parsimonious models

Fenton and Karma (1998) presented a minimal ionic model with three membrane currents (controlled by three gating variables) which was able to reproduce the restitution and spiral wave behavior of more complex AP models. The simplicity of the model means that it is readily adaptable to reproduce the activity of new models and cell types (Oliver & Krassowska, 2005; Podziemski & Żebrowski, 2013; Whittaker, Benson, Teh, Schneider, & Colman, 2019). However, although the Fenton and Karma (1998) model was able to reproduce APD and CV restitution curves, it could not reproduce different AP shapes, such as a “spike‐and‐dome” morphology, accurately. By adding a fourth variable to the three membrane currents, Bueno‐Orovio et al. (2008) created a minimal ventricular human model which elucidated the minimal physiological requirements necessary to reproduce realistic AP morphologies, in addition to APD and CV restitution curves. The model of Bueno‐Orovio et al. (2008) has subsequently been adapted to recapitulate human atrial (Lenk et al., 2015) and pulmonary vein electrophysiology (Green, Thomas, & Terry, 2017), where it has been shown to be capable of generating accurate spatiotemporal excitation patterns found in anatomical and spiral wave reentry (Richter, Lind, Seemann, & Maass, 2017).

Gray and Pathmanathan (2016) presented a “parsimonious” rabbit AP model, by combining a Hodgkin–Huxley model of fast sodium current, *I*_Na_, with a phenomenological model of repolarization. Calibration of the *I*_Na_ model involved an innovative use of dynamic current–voltage (*I*–*V*) relations during the AP upstroke, which is driven predominantly by *I*_Na_ and thus contains information regarding its kinetics. By coupling the *I*_Na_ model with a phenomenological model of repolarization, given by $I_{K}=g_{K}e^{-b\left(V_{m}-E_{K}\right)}\left(V_{m}-E_{K}\right)$, where *b* is a parameter that controls AP shape, it was shown that the parsimonious model was able to elucidate the minimal physiological requirements for important emergent phenomena such as alternans at fast rates and spiral wave break. The fact that the parameters of the parsimonious model have physiological meaning and are identifiable makes it an attractive choice for producing generalizable mechanistic insights, and raises questions about the level of detail required for making predictions about mechanisms of tissue‐level behaviors using cardiac models. This is especially important when considering how to tailor AP models toward specific patients, as the clinical data are likely to be too limited to enable parameterization of more complex models.

### 4.5 Patient‐specific models

In Section 4.3, different approaches toward creating cell‐specific models of cardiac electrophysiology were discussed. However, with the coming era of precision medicine, the idea of *patient*‐specific cardiac electrophysiology models is a tantalizing possibility. At present, fully personalized models, that is, those in which *every* parameter is patient‐derived, remain unattainable (Clerx, 2018). However, several advances have been made in tailoring electrophysiology models to reproduce clinical electrophysiological measurements from individual patients, which, while not “fully” personalized, may nonetheless be able to contribute to the diagnosis and interpretation of cardiac conditions, as well as decision making within a clinical setting. We here focus only on attempts to tailor cellular electrophysiology toward individual patients; for a review which includes personalized *excitation* models see Gray and Pathmanathan (2018).

The use of patient‐specific anatomies derived from MRI and CT imaging data in cardiac modeling is now relatively widespread, and forms an essential aspect of many clinically oriented cardiac modeling pipelines (Fastl et al., 2018; Prakosa et al., 2018). Current clinical imaging technologies can uncover regions of fibrosis, ischemia, and post‐infarct scar zones—by assigning different electrophysiological properties to these regions in subject‐specific anatomies, the models have in some sense a degree of “personalized” electrophysiology (Arevalo et al., 2016; MacLeod et al., 2009). However, some studies have gone beyond this level of personalization and have attempted to calibrate models to reproduce specific electrophysiological properties of individuals measured in a clinical setting. For example, Potse et al. (2014) demonstrated that parameters of the ten Tusscher et al. (2004) human ventricular model could be tuned to reproduce measured ECGs and endocardial electrograms from two different patients with heart failure and left bundle branch block. This calibration involved a substantial reduction in *I*_K1_ and introduction of a linear gradient in *I*_Ks_ in order to best match ECGs, which nonetheless showed some persistent model mismatch (the deep S waves could not be reproduced in simulations). Such model findings are useful in that they reveal either incomplete mechanistic understanding of how a disease works, significant model inadequacy which requires attention, or likely both.

Existing models of the human atrial AP rarely recapitulate clinical features of AF in a given patient, given their generic nature. Lombardo, Fenton, Narayan, and Rappel (2016) created human atrial models which were tailored toward specific patients by simultaneously optimizing the parameters of a detailed and simplified model to recapitulate clinical mapping measurements of AP morphology, as well as APD and CV restitution curves, for five patients undergoing ablation therapy. For both levels of detail, optimized parameter sets were able to recapitulate clinical data well, but were notably different from original published parameters and between patients, highlighting the benefits of patient‐specific adjustment. Spiral waves with similar trajectories in each patient were produced by both models, suggesting that simplified models may be a better choice for simulations of cardiac electrophysiology in spatially extended domains, due to their increased computational efficiency (not to mention advantages in terms of parameter identifiability, see Johnstone, Chang, et al., 2016).

Corrado et al. (2017) presented a method for constructing personalized human atrial electrophysiology models from multi‐electrode catheter measurements, using the Mitchell and Schaeffer (2003) minimal ionic model. The method was applied to five clinical datasets recorded from AF patients, where models were fitted with a ∼5% error in restitution curves in a matter of minutes, representing a relatively robust and rapid approach. In Corrado et al. (2018), the atrial anatomy was recorded with an electroanatomical mapping system in seven patients, and then an S1–S2 stimulation protocol was applied from the coronary sinus and high right atrium, which served as independent training and validation datasets, while recording activation patterns across the atrium. Parameters of a modified ionic model robust to pacemaking behavior (Corrado & Niederer, 2016) were fitted to CV restitution curves and estimated local ERP, then interpolated across each atrium. Personalized models recapitulated S1–S2 activation times from the coronary sinus, and were able to predict activation times from the independent high right atrium recordings.

## 5 AVOIDING COMMON PITFALLS

There are a number of pitfalls associated with calibration of cardiac electrophysiology models, largely relating to the concepts discussed in Section 2. However, most of these can be identified using synthetic data studies before attempting to calibrate to real data (as we did in the example of Figure 3), avoiding the discovery of such pitfalls on a post hoc basis, and the subsequent potential need to redo challenging experiments or rerun costly parameter optimization routines. Here we discuss some of the most common pitfalls, and suggest ways to avoid them.

### 5.1 A priori unidentifiability

In some cases, it is mathematically certain that parameters are unidentifiable, as we can tell simply by examining the equations. A case that occurs commonly in cardiac electrophysiology models is within ion‐channel gating (Fink & Noble, 2009). For historic/graphical fitting reasons, it is reasonably common to see gating rates written as:

(3) $$k\;=A\exp\left(\frac{V-B}{C}\right),$$

where *k* is a rate, *V* is membrane voltage, and *A*, *B*, and *C* are constant parameters. This can be rewritten as

(4) $$\begin{array}{ll} & k=A^{\prime }\exp\left(B^{\prime }V\right). \end{array}$$

where *A* ^′^ = *A*exp(−*B*/*C*) and *B* ^′^ = 1/*C*. As a result, there exist infinite combinations of *A* and *B* which generate the same value of *A* ^′^.

To some extent this redundancy does not pose a problem (as long as parameters *A* and *B* do not appear elsewhere and not are not interpreted as meaningful values) because, given *A* ^′^ has been fitted, the same rate *k* will be predicted for any values of *A* and *B* consistent with *A* ^′^. But we believe this situation should be avoided as allowing an optimization algorithm to attempt to fit all three parameters is making the problem far more difficult than necessary: an extra parameter adds another dimension to the search space; and an a priori unidentifiability creates a flat line (in a 2D optimization), surface (3D), or manifold (>3D) on which an optimization algorithm will spend considerable effort failing to converge as it attempts to find a (nonexistent) minimum.

### 5.2 Finding the global minimum

Even when parameters are theoretically identifiable from a given dataset (and there is therefore one global minimum to find) it is not an easy task. A whole field of optimization algorithm design exists. Here we will discuss a few aspects that we have found invaluable to consider.

#### 5.2.1 The importance of parameter transforms

**Figure 8:**
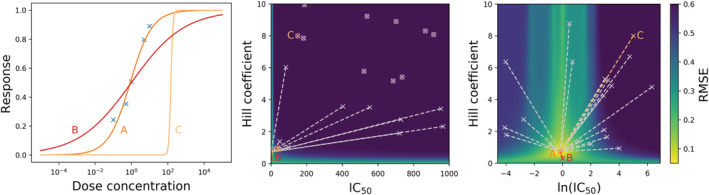
Improved optimization with parameter transforms. Left: blue crosses indicate some dose–response data points in arbitrary units. The orange line represents the best fit with smallest root‐mean‐square error (RMSE). The yellow and red lines indicate possible different initial guesses. Middle: untransformed parameter space—a heatmap of RMSE for across a wide range of parameters. Twenty initial guesses (gray crosses) and final optimization locations (orange/purple circles) are shown linked with dashed lines. Only half of the initial guesses reach the global optimum with the others becoming stuck (purple circles) due to the optimizer seeing a “flat” surface. Right: transformed parameter space (ln [IC_50_])—in this case all 20 initial guesses sampled across this space reach the global minimum

In Figure 8, we show a very simple example which highlights the need to consider performing optimization in a transformed parameter space. We consider the apparently simple task of fitting a dose–response or Hill curve to some data points. The equation for a dose–response curve is

(5) $$\text{Response}=\frac{1}{1+{\left(\frac{{\mathrm{IC}}_{50}}{\text{Dose}}\right)}^{\text{Hill}}},$$

with two parameters, “IC_50_” the concentration at which the response is 50% of control, and “Hill” a coefficient dictating the steepness of the dose–response curve.

Figure 8 shows how considering IC_50_ untransformed using a Nelder and Mead (1965) simplex algorithm leads to difficulties—optimization from many initial guesses gets “stuck” and fails to find the global minimum. In contrast, if we use a log scaling and instead allow the optimization algorithm to work with the parameter set **θ** = {ln(IC_50_), Hill} we see how optimization from initial guesses across the same range of values succeeds every time. In this case, the transform is somewhat evident by considering the log‐scaling convention used to plot dose–response curves.

For ion‐channel kinetics, state transition rates *k* can be written as *k* = *A*exp(*BV*) or sometimes *k* = exp(*C* + *DV*), where *A*, *B*, *C*, *D* are parameters and *V* is voltage. In these cases, scalings of **θ** = {ln(*A*), *B*} work well (Beattie et al., 2018) or, equivalently, an untransformed **θ** = {*C*, *D*} (Teed & Silva, 2016). A detailed comparison of optimization on transformed parameter spaces in ion‐channel calibration can be found in the supplement to Clerx, Beattie, et al. (2019), as well as some advice on constraints for these rates.

For ion current densities (e.g., ion‐channel maximal conductances, “*g*”) it is common to use untransformed parameters. We have found it can be beneficial to use a transform of $\theta =\log g/\log\hat{g}$ (with inverse $g={\hat{g}}^{\theta }$), where $\hat{g}$ is a positive number representing an initial guess or representative size for the maximal conductance. This transform has two benefits: firstly, it has a scaling property such that an optimizer exploring equally either side of the initial guess (*θ* ± *a*) corresponds to operations such as “doubling” (+ 100%) and “halving” (− 50%) $\hat{g}$, which is useful due to similar magnitude effects on APs from these changes (https://mirams.wordpress.com/2015/06/08/triangles/); and secondly, it constrains the conductance to be positive regardless of where the optimizer explores. In general, transforms such as these can be useful to enforce positivity (or other) constraints when using unconstrained optimization algorithms (Hass et al., 2019; Raue et al., 2013).

#### 5.2.2 Testing robustness

The example in Figure 8 also highlighted the importance of running optimizations from multiple initial guesses. It is important to gain some confidence that you are reliably finding the same global minimum/posterior from different initial guesses, and that adding more initial guesses and runs is not going to find a new and better optimum.

Similarly, it can be useful to try different optimization/inference algorithms as some are more suited to particular calibration challenges than others. In the same way that adding extra start points builds confidence in having found the global optimum, getting the same result from different algorithms is reassuring too. For this it can be helpful to use a software library of routines such as those available in R, SciPy (Jones, Oliphant, Peterson, et al., 2001), or PINTS (Clerx, Robinson, et al., 2019).

#### 5.2.3 Visualizing the optimization surface

Information about the difficulty of the optimization can be found by performing evaluations of the objective/likelihood function along lines (1D) or slices (2D) in parameter space around areas of interest, particularly the parameter sets in synthetic data studies and final results of a real calibration exercise. The plot in Figure 8 is an example of this, where it is cheap enough to evaluate the function across a wide range of parameters to build up the heatmaps. This exercise can highlight whether there are likely to be multiple local minima, and allows you to check how smooth the function is and can also inform appropriate transforms which make the target convex and easy to minimize (Hass et al., 2019). We have also found examples where this approach highlights numerical issues, discussed in the next section.

#### 5.2.4 Convergence of numerical solutions

Most computational scientists will be familiar with the concept of convergence of numerical solutions—as the numerical solution is made more accurate the change in the results should decrease. In the setting of numerical solution of ODEs it is usual to pick solver tolerances or timesteps based on examining convergence of a solution over time with a given set of parameters, and deciding when an accurate enough solution is obtained.

It is easy to miss the concept of convergence *with respect to parameters*: when calibrating models the numerical evaluation of the likelihood/objective function must have converged as well. Standard least squares techniques with time series sampled at a high rate can require a more refined numerical scheme than might be used for a single forward simulation (see, e.g., Figure 3 in Clerx, Beattie, et al., 2019).

To summarize this section, there are many potential pitfalls and things that can go wrong in model calibration, but many of these can be spotted with synthetic data and practical identifiability assessments.

## 6 CHALLENGES AND OPEN QUESTIONS

There are a lot of challenges and open questions involved in calibration of cardiac models. In this section, we highlight a number of areas that deserve further research.

### 6.1 Sources of variability

Biological variability occurs at all scales. Depending on the question, we may need to consider stochastic opening of ion channels, genetic variability and mutations, gene‐regulatory network activity, regulation of ion‐channel expression in the membrane, cell geometry, spatial heterogeneity in these things throughout tissue regions, up to differences between individuals across different patient populations. Perhaps more dauntingly, most of these things will correlate with and influence one another. While we have reviewed some statistical methods for dealing with variability, measuring and accounting for all these inter‐cell, inter‐region, and inter‐individual variabilities and establishing which ones are important for understanding fundamental biological mechanisms or particular clinical approaches is extremely challenging and will almost certainly require experimental, simulation and statistical advances. Another important point is that variability in experimental recordings may not be due to real biological variability, but experimental artifacts.

#### 6.1.1 Experimental noise and artifacts

Biological systems are notoriously difficult to measure and control, so that any experiment should be expected to contain a degree of noise. This can include Gaussian noise on a time trace but also systematic bias and transient “artifacts,” and accounting for these issues is another challenge in model calibration. We will briefly review difficulties in patch‐clamp measurements, which are a common source of data for calibration, but similar considerations arise when using fluorescence to measure voltage and calcium signals, in accounting for buffering of dyes, binding/reaction time rates and calibrating the magnitude of responses.

Some issues arise from the fact that cells are active, living things that are impacted by (and react to) isolation and subsequent experiment. For instance, channel expression levels in isolated ex vivo cardiac myocytes are known to be affected by the isolation procedures (Rajamani et al., 2006; Yue et al., 1996). In this sense, even an isolated native human myocyte should be regarded as a model system. Methods to account for experimental conditions in calibration were discussed in Section 4.1.2, while Section 4.1.4 discusses the problem of translating between cell types.

For ion current measurements in particular, it can be difficult to isolate the phenomenon of interest, that is, to separate the current under investigation from leak and capacitative currents, other native currents in myocytes, or endogenous currents in expression systems. Some classic methods to isolate currents are separating them via their kinetics, subtracting a drug‐blocked protocol, and P/N leak subtraction (Bezanilla & Armstrong, 1977), while recent advances include the use of dynamic clamp (Milescu et al., 2008) and the “sequential dissection” protocol (Banyasz et al., 2011; for a further review of recent advances see Bebarova, 2012).

Another source of problems is lack of control over the experimental system. For example, there are several factors in voltage‐clamping that can cause a difference between the intended and the true membrane potential (Lei, Clerx, Whittaker, et al., 2019; Neher, 1995; Sherman et al., 1999; White, Sekar, & Kay, 1995). Similarly, intracellular ion concentrations are often assumed to be set by the pipette concentrations, but can vary in nontrivial ways (Mathias, Cohen, & Oliva, 1990), which will also affect calculated estimates of reversal potentials.

Studies of identifiability and calibration techniques tend to start from an assumption of perfect data, or data contaminated only by Gaussian noise. Extending these studies to include more realistic noise models will be a significant challenge for the future. Some simple measures can be taken, for example, Clerx, Collins, and Volders (2015) modified the identifiability analysis by Fink and Noble (2009) to omit parts of the data routinely “cut out” of current recordings to avoid capacitative artifacts. But in general a more complex approach will be required, including modeling the origins of experimental artifacts as was recently demonstrated in Lei, Clerx, Whittaker, et al. (2019). In this respect it is interesting to notice that any compensation for noise or artifacts carried out experimentally introduces new parameters into the statistical noise model (see Section 2.4), which may then need to be estimated. For the best results, modelers should get closely involved in the experimental process, so that experiments can be designed and carried out with model calibration in mind.

### 6.2 Model discrepancy

“All models are wrong,” as the common aphorism in statistics goes. Indeed, all models are imperfect representations of reality, and do not perfectly represent the real system that generated the data we observe (even with the best possible parameterization). The difference between the real system and the model is termed the “model discrepancy” or “model mismatch.” This difference can be due either to the limitations of our understanding of the system, or due to our purposeful abstractions and simplifications. However, credibility of model predictions and model parameters relies on how accurately the model represents the true system.

Model discrepancy is difficult to characterize and is an active area of statistical research. There are some approaches which include knowledge of the model discrepancy into the inference/calibration process for parameters, adding a specific term for discrepancy into Equation (1). For example, Kennedy and O'Hagan (2001) fit a Gaussian Process for the model discrepancy at the same time as inferring the model parameters. The first efforts we have seen to apply this approach to ion‐channel kinetics were published by Plumlee, Joseph, Yang, Roshan Joseph, and Yang (2016). However, there can be problems of identifiability with this approach so good priors are needed for the discrepancy terms (Brynjarsdóttir & O'Hagan, 2014), and there is no obvious method to forecast discrepancy in new situations. An alternative method by Lyddon, Walker, and Holmes (2018) is to use a Bayesian nonparametric learning approach to relax the assumption that the model is true, which aims to correct the posterior of the model parameters.

In cardiac electrophysiology modeling it is common for existing kinetic models of particular currents to be re‐used and combined into a new AP model (Niederer et al., 2009). The extent to which our estimates of current densities in the new model are vulnerable to error due to any discrepancies in the existing kinetic models remains unclear; as do the upstream effects of discrepant kinetics on predictions. Model discrepancy and model selection are related questions, as each possible model structure will have a different discrepancy. A number of studies have drawn attention to this issue and begun to investigate it (Fabbri, Goversen, Vos, van Veen, & de Boer, 2019; Ravagli et al., 2016).

It is often difficult to establish whether discrepancy between model fits or predictions and data is due to experimental artifacts/limitations, model discrepancy, or both (Lei, Clerx, Whittaker, et al., 2019). Characterizing the size and consequences of model discrepancy is a major and fundamental challenge for our field as predictions begin to be used in safety‐critical settings. An extensive perspective review on modeling discrepancy during model calibration is carried out by Lei et al. (2020).

### 6.3 Model selection

As cardiac electrophysiology models are not generally derived from first principles, there is usually more than one set of equations describing the biological system which could be used. Choosing which set of equations to use (rather than just calibrating the parameters within them) is termed “model selection.” Model selection can have important consequences when it comes to making predictions, as different models have typically been calibrated/validated using different experimental data, and thus differ in complexity, the degree of parameter identifiability, and will exhibit different discrepancy in different situations.

There is no clear procedure for selecting which model to use in which context, and small model discrepancy against the training data is often prioritized over parameter identifiability and discrepancy in distinct validation, which may lead to spurious predictions in new situations (see Figure 2). Furthermore, when it comes to modeling certain phenomena, such as drug binding, it may be preferable to select the model “on the fly” as part of the model calibration process based on the data, as the model required depends on the drug block mechanism (Perissinotti et al., 2015). At present, automating or clearly identifying a procedure for the model selection process is something of an open challenge.

Studies which benchmark different models of the same system can be considered when choosing a model (Bett, Zhou, & Rasmusson, 2011; Cherry & Fenton, 2007; Elshrif & Cherry, 2014; Verkerk & Wilders, 2013; Wilhelms et al., 2013), as these typically highlight the strengths and weaknesses of different models with regards to different contexts of use and the modeling of specific phenomena.

An alternative approach to model selection is to optimize the structure of a model at the same time as the parameters, which has been described for neuronal (Menon, Spruston, & Kath, 2009) and cardiac (Teed & Silva, 2016) ion‐channel models. Methods of model selection which embrace multiple models and thus reduce uncertainties and biases associated with using a single model are ensemble approaches or weighted ensembles of different model structures (e.g., Bayesian model averaging—see Fragoso, Bertoli, and Louzada, 2018 for a review). To our knowledge, this has not yet been used in cardiac electrophysiology modeling. An important line of research would be design of experiments to perform both model selection *and* parameterization at the same time.

### 6.4 Sharing and reproducibility

The history of cardiac electrophysiology modeling has been one of “iterative interaction between experiment and simulation” (Noble & Rudy, 2001), enabled by a close cooperation between modelers and experimenters. Its future is being shaped by projects like the IUPS Physiome Project, which aims to create the databases and modeling frameworks needed to build hierarchical, modular, multiscale models of human physiology (Bassingthwaighte, 2000; Hunter, Robbins, & Noble, 2002). Progress is made when researchers can build on each other's work, reusing data and models freely, thoroughly inspecting and replicating each other's findings. As calibration is a vital step in modeling, we argue that the entire calibration process must be reproducible and open to all to inspect, modify and rerun. Providing a framework to do so is a goal of the Cardiac Electrophysiology Web Lab project (Daly et al., 2018).

There are several aspects to reproducible calibration, including sharing of data (and detailed meta‐data), sharing of models, and sharing of any code used in calibration, including software for simulation, optimization, and statistical inference. Sharing of models and open source simulation/optimization tools is well established (see Clerx et al., 2016; Clerx, Robinson, et al., 2019; Hedley, Nelson, Bellivant, & Nielsen, 2001; Mirams et al., 2013; Yu et al., 2011), and it is increasingly common for papers involving simulation or calibration to post their code on publicly accessible repositories (see, e.g., the GitHub repository accompanying this manuscript).

Sharing of data (and meta‐data) suitable for model calibration has been slower to develop. Firstly, as studies like Willms et al. (1999) and Clerx, Beattie, et al. (2019) show, publishing tables of “summary data” such as time constants and steady states is insufficient for reliable calibration, and instead digital sharing of larger data sets (e.g., time‐traces) is required. One way to facilitate such data sharing would be the creation of a common data format and a centralized repository, and this approach has been highly successful in other fields, for example, the sharing of ECGs in PhysioBank (Goldberger et al., 2000). An interesting step in this direction has recently been taken by the Channelpedia project, which has begun storing ion current traces (Ranjan et al., 2011, 2019), but does not yet facilitate user uploads. However, there can be substantial costs associated with hosting and providing access to large data sets, so that a more decentralized approach to data sharing might also be considered. For a further discussion of these issues, see also Cannon and D'Alessandro (2006) and Fink, Niederer, et al. (2011).

In addition to data, there must be enough meta‐information (including any voltage‐clamp or stimulus protocols) to enable experimental replication, but also in silico replication as an automated virtual experiment (Cooper, Vik, & Waltemath, 2015). In terms of implementations of virtual experiments, the SED‐ML language provides a basic framework to describe them (Waltemath et al., 2011) with more flexible “protocol” descriptions (which also include postprocessing and can be applied to any model using shared ontology terms) provided by the “functional curation” language in the Cardiac Electrophysiology Web Lab (Cooper, Mirams, & Niederer, 2011; Cooper, Scharm, & Mirams, 2016). Converging these efforts with standards such as MICEE (Quinn et al., 2011) so that a lab's experiments can be re‐run as virtual experiments will be an important step.

While long‐term solutions to these problems may require large‐scale community effort, we urge both modelers and experimenters to view publication of their models, data sets, and software not as “supplementary” to a manuscript, but as the main product: a building block in the greater scientific endeavor.

## 7 CONCLUSIONS

This century has seen important advances in the methods and applications pertaining to the calibration of ionic and cellular models of cardiac electrophysiology, many of which have been outlined in this review. Nonetheless, we have also highlighted open challenges which remain, as well as common pitfalls associated with model calibration, which we suggest can often be identified and appropriately mitigated through synthetic data studies and improved standards when it comes to reporting and availability of experimental data.

## CONFLICT OF INTEREST

The authors have declared no conflicts of interest for this article.

## AUTHOR CONTRIBUTIONS

**Dominic G. Whittaker:** Conceptualization; formal analysis; investigation; methodology; project administration; writing‐original draft; and writing‐review and editing. **Michael Clerx:** Conceptualization; investigation; methodology; software; supervision; writing‐original draft; and writing‐review and editing. **Chon Lok Lei:** Formal analysis; investigation; methodology; resources; software; writing‐original draft; and writing‐review and editing. **David Christini:** Conceptualization; funding acquisition; investigation; methodology; and writing‐review and editing. **Gary Mirams:** Conceptualization; funding acquisition; investigation; methodology; project administration; supervision; writing‐original draft; and writing‐review and editing.

## DATA AVAILABILITY STATEMENT

Python code for the examples in this paper is openly available at https://github.com/CardiacModelling/WIRES. A permanently archived version is available on Zenodo at doi:10.5281/zenodo.3523199.

## RELATED WIREs ARTICLES

Integrative modeling of the cardiac ventricular myocyte

Modeling calcium regulation of contraction, energetics, signaling, and transcription in the cardiac myocyte
